# Analysis of the giant genomes of *Fritillaria* (Liliaceae) indicates that a lack of DNA removal characterizes extreme expansions in genome size

**DOI:** 10.1111/nph.13471

**Published:** 2015-06-08

**Authors:** Laura J. Kelly, Simon Renny‐Byfield, Jaume Pellicer, Jiří Macas, Petr Novák, Pavel Neumann, Martin A. Lysak, Peter D. Day, Madeleine Berger, Michael F. Fay, Richard A. Nichols, Andrew R. Leitch, Ilia J. Leitch

**Affiliations:** ^1^ School of Biological and Chemical SciencesQueen Mary University of LondonLondonE1 4NSUK; ^2^ Jodrell LaboratoryRoyal Botanic GardensKewRichmondTW9 3DSUK; ^3^ Department of Plant SciencesUniversity of California DavisDavisCA95616USA; ^4^ Biology Centre CASInstitute of Plant Molecular BiologyCZ‐37005České BudějoviceCzech Republic; ^5^ Plant Cytogenomics Research GroupCEITEC – Central European Institute of TechnologyMasaryk UniversityKamenice 5CZ‐62500BrnoCzech Republic; ^6^ School of Biological and Biomedical SciencesDurham UniversitySouth RoadDurhamDH1 3LEUK; ^7^ Rothamsted ResearchWest CommonHarpendenHertfordshireAL5 2JQUK

**Keywords:** DNA deletion, *Fritillaria*, genome size evolution, genome turnover, Liliaceae, repetitive DNA, transposable elements (TEs)

## Abstract

- Plants exhibit an extraordinary range of genome sizes, varying by > 2000‐fold between the smallest and largest recorded values. In the absence of polyploidy, changes in the amount of repetitive DNA (transposable elements and tandem repeats) are primarily responsible for genome size differences between species. However, there is ongoing debate regarding the relative importance of amplification of repetitive DNA versus its deletion in governing genome size.
- Using data from 454 sequencing, we analysed the most repetitive fraction of some of the largest known genomes for diploid plant species, from members of *Fritillaria*.
- We revealed that genomic expansion has not resulted from the recent massive amplification of just a handful of repeat families, as shown in species with smaller genomes. Instead, the bulk of these immense genomes is composed of highly heterogeneous, relatively low‐abundance repeat‐derived DNA, supporting a scenario where amplified repeats continually accumulate due to infrequent DNA removal.
- Our results indicate that a lack of deletion and low turnover of repetitive DNA are major contributors to the evolution of extremely large genomes and show that their size cannot simply be accounted for by the activity of a small number of high‐abundance repeat families.

## Introduction

Genome size may differ by > 40‐fold between species of the same ploidy within a single genus of plants (Bennett & Leitch, 2012; Kelly *et al*., 2012). The observation that a few families (Hawkins *et al*., 2006; Piegu *et al*., 2006), or even a single family (Neumann *et al*., 2006), of transposable elements (TEs) can dominate plant genomes and account for variation in genome size between closely related species has led to the suggestion that differences in the propensity for TE amplification play a primary role in governing genome size change (Grover & Wendel, 2010). However, at least some plant and animal species with large genomes appear to lose DNA more slowly than those with smaller genomes (Bensasson *et al*., 2001; Wicker & Keller, 2007; Hawkins *et al*., 2009; Hu *et al*., 2011; Sun *et al*., 2012a), indicating that differences in the rate of DNA removal may play an important role in determining genome size. Recombination‐based mechanisms, such as illegitimate recombination and unequal intrastrand homologous recombination, can delete substantial amounts of DNA (Ma *et al*., 2004) and comparatively high rates of deletion in smaller genomes may result in a dearth of ancient TE copies, as amplified DNA is rapidly purged (Wang & Liu, 2008; Hawkins *et al*., 2009; The International Brachypodium Initiative, 2010; Blass *et al*., 2012). Nevertheless, it has also been argued that variation in DNA removal rate is unlikely to be the major determinant of genome size (Vitte & Bennetzen, 2006), and the significance of differences in the efficiency of DNA deletion in governing genome expansions remains unclear.

If the general view that plant genomes expand as a result of increased activity of relatively few repeat families (Vitte & Bennetzen, 2006; Elbaidouri & Panaud, 2013; reviewed by Bennetzen & Wang, 2014) holds true, differences between species with extremely large genome sizes should also be explained by the proliferation of a small number of highly abundant repeats. To test this prediction, we analysed the repeat content of species of *Fritillaria*, a genus in the lily family (Liliaceae; monocotyledons) that represents the most extreme case known in plants of absolute genome size expansion independent of recent whole‐genome duplication. Reported genome size (1C) values in diploid *Fritillaria* vary between 30.15 and 85.38 Gb (Leitch *et al*., 2007; Ambrožová *et al*., 2011). This *c*. 55 Gb difference equates to > 350 times the size of the *Arabidopsis thaliana* genome (Bennett *et al*., 2003; Bennett & Leitch, 2012) and > 860 times that of *Genlisea aurea*, the smallest land plant genome sequenced to date (Leushkin *et al*., 2013). Assuming a similar amount of non‐TE‐related genes as in other monocotyledon genomes, such as banana (*Musa acuminata*) (139 Mb; D'Hont *et al*., 2012) and rice (*Oryza sativa*) (101 Mb; International Rice Genome Sequencing Project, 2005), < 1% of *Fritillaria* genomes would comprise protein‐coding gene sequences. Moreover, genome sizes in excess of 50 Gb appear to have arisen independently in separate lineages of *Fritillaria* (Leitch *et al*., 2007; Ambrožová *et al*., 2011), providing an unparalleled opportunity to examine replicated expansions near the upper end of the genome size scale.

## Materials and Methods

### Taxon sampling and plant material

We selected *Fritillaria affinis* (Schultes) Sealy and *Fritillaria imperialis* L. to test the hypothesis that differences in genome sizes between species are governed by differential amplification of a small number of highly abundant repeats. Each species represents one of two major species groups within *Fritillaria* (Rønsted *et al*., 2005; Supporting Information Fig. S1) and has a genome size of *c*. 45 Gb (Ambrožová *et al*., 2011; Table S1). Low‐pass 454 sequencing was conducted on eight further species (Fig. S1); these were included so that repetitive elements that have amplified specifically in *F*.* affinis* or *F*.* imperialis* could be identified. Species were selected to represent different *Fritillaria* subgenera (Rix, 2001), and its sister group *Lilium* (Rønsted *et al*., 2005), and to span the range of known genome sizes in the genus (Leitch *et al*., 2007; Ambrožová *et al*., 2011). For phylogenetic reconstruction of evolutionary relationships and inference of ancestral genome size, we used an expanded set of species (Table S2) including members of all eight subgenera of *Fritillaria*, and representatives of related genera within the Liliaceae (Fig. S1).

### DNA extraction

Total genomic DNA was extracted from fresh or silica‐dried leaves using a cetrimonium bromide (CTAB) method modified from Doyle & Doyle (1987) with purification via CsCl density‐gradient ultracentrifugation. Alternatively, existing DNA was taken from the DNA bank at the Royal Botanic Gardens, Kew (Table S2).

### Chromosome counts

Chromosome counts were conducted to verify ploidy of individuals used for 454 sequencing and genome size estimation. Young roots were pretreated with saturated alpha‐bromonaphthalene at 4°C for 24 h, fixed in ethanol : glacial acetic acid (3 : 1) at 4°C for 48 h, and stored in 70% ethanol at −20°C. Roots were washed in double distilled water at room temperature (RT) for *c*. 30 min, hydrolysed in 1 M HCl at 60°C for 4–12 min, and stained with Schiff's reagent for ≥ 30 min at RT. Root tips were squashed in 45% acetic acid and counterstained with 2% aceto‐orcein as necessary. Where suitable root material was unavailable, we used published counts from the same accessions (Leitch *et al*., 2007; Ambrožová *et al*., 2011). Where counts could not be obtained for accessions used for genome size estimation, ploidy was inferred by comparing the 1C values with published measurements from the same, or closely related, species with known ploidy (Table S3).

### Genome size estimation by flow cytometry (FC)

We used FC to estimate nuclear DNA contents of all species included in 454 sequencing (Fig. S1) and for species included in phylogenetic analyses, where previously published FC estimates were not available. Samples were prepared and analysed as described in Pellicer *et al*. (2014); *Pisum sativum* (‘Ctirad’; 1C = 4445 Mb; Doležel *et al*., 1998) and *Allium cepa* (‘Ailsa Craig’; 1C = 16405 Mb; Van't Hof, 1965) were used as internal standards. For each individual analysed, three samples were prepared (from separate leaves or different parts of the same leaf) and three replicates of each sample run. Fresh leaf material was unavailable for *Fritillaria pluriflora*, and therefore a value estimated previously for the same accession with Feulgen microdensitometry (FM) was used (Table S1).

### Phylogenetic analysis of species relationships

Relationships between *Fritillaria* species and related genera were reconstructed from a combined data set comprising sequences from three plastid genome regions: *c*.1.6 kb of the maturase K (*mat*K) gene, *c*.1.4 kb of the ribulose‐1,5‐bisphosphate carboxylase/oxygenase large subunit (*rbc*L) gene and *c*.1.4 kb of the ribosomal protein L16 (*rpl*16) gene (partial intron and 3ʹ exon). Sequences were either taken from Day *et al*. (2014) or amplified and sequenced as described in Day *et al*. (2014). Sequences used in phylogenetic analyses have been submitted to GenBank (accession nos. KP998197–KP998208; see Table S2). As a consequence of low levels of variation, sequences were aligned manually using macclade v4.04 (Maddison & Maddison, 2002); indels within coding regions were aligned so as to maintain the correct reading frame. Phylogenetic analyses were conducted using maximum parsimony and Bayesian inference (BI). Maximum parsimony analyses were conducted in PAUP* v4.0 b10 (Swofford, 2003) as described in Kelly *et al*. (2013). For phylogenetic analyses using BI, best‐fit models of evolution for each data set were selected with the Akaike Information Criterion in mrmodeltest v2.3 (Nylander, 2004). Data sets were partitioned into separate codon positions for *mat*K and *rbc*L and codon positions and intron for *rpl*16; model testing was carried out on each partition separately. Analysis by BI was carried out using mrbayes v3.2.1 (Ronquist *et al*., 2012) as described in Kelly *et al*. (2013). Parameter values from each run were viewed in tracer v1.5 (Rambaut & Drummond, 2009) to confirm that effective sample sizes of > 200 had been obtained for each parameter and stationarity reached. Trees sampled during the first 500 000 generations of each run were discarded as the burn‐in. A majority rule consensus tree showing all compatible groupings was constructed using bayestrees v1.3 (www.evolution.reading.ac.uk/BayesTrees.html) (Fig. S1). The combined alignment and phylogenetic trees have been submitted to TreeBase (study accession: S16132).

### Ancestral genome size reconstruction

**Figure 1:**
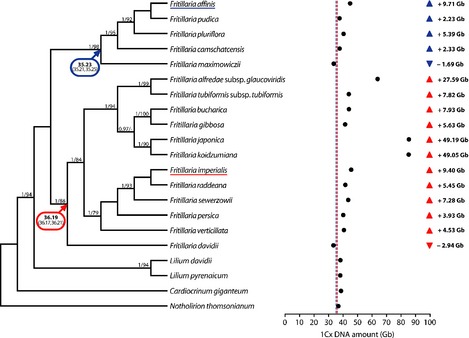
Ancestral genome size reconstruction and evidence for genome expansion in *Fritillaria*. Phylogenetic relationships between species of *Fritillaria* and related genera are shown; values above branches indicate node support (posterior probabilities of ≥ 0.95/bootstrap percentages ≥ 70). Ancestral genome sizes for the most recent common ancestor (MRCA) of each major *Fritillaria* clade are shown; 95% confidence intervals are given in parentheses. Closed circles indicate monoploid genome size (1Cx‐values in Gb) for extant species; dashed lines indicate the ancestral genome sizes for the MRCA of the *F*.* affinis* (blue) and *F*.* imperialis* (red) clades. For each species of *Fritillaria*, the increase or decrease in genome size relative to the MRCA of its clade is indicated.

To test for evidence of independent genomic expansions in *F*.* affinis* and *F*.* imperialis*, we conducted ancestral genome size reconstruction using genome size data listed in Table S1. To remove the effect of genome size increase resulting from recent polyploidization, monoploid genome size (1Cx; Greilhuber *et al*., 2005) values were used; 1Cx‐values were calculated by dividing the 2C value by the ploidy level (Table S3). Ancestral genome sizes were reconstructed for the most recent common ancestor (MRCA) of the clades containing *F*.* affinis* and *F*.* imperialis* (Figs 1, S1) using bayestraits v1.1beta by analysing genome sizes for extant species (1Cx‐values in Gb; Table S1) as continuously varying data (Pagel, 1997, 1999) along with the 36 000 post burn‐in trees from the mrbayes analysis. The 1Cx‐values from *Fritillaria* and relatives have a distribution that is significantly different from normal (one‐sample Kolmogorov–Smirnov test of untransformed data; *P *=* *0.008). Therefore, before analysis they were Box‐Cox transformed using the bcPower function in the ‘car’ package of R (R Core Team, 2014) with a lambda setting of −2 (one‐sample Kolmogorov–Smirnov test of transformed data; *P *=* *0.443).

The best‐fit model for analysis of continuously varying characters (i.e. random walk versus directional) was selected by conducting Bayes factor tests using the logarithm of the harmonic mean estimated from five separate runs of bayestraits (as outlined in the bayestraits manual; www.evolution.rdg.ac.uk/Files/BayesTraits-V1.0-Manual.pdf) under the Markov Chain Monte Carlo option, with the following settings: 1000 million iterations, burn‐in of 250 million iterations, sampling every 10 000 generations, estimating the scaling parameters (δ, κ and λ) and with the RateDev (RD) parameter optimized to maximize the number of iterations with the recommended 20–40% acceptance rate. The directional model was favoured in the majority of iterations and was therefore selected, and the posterior distribution of model parameters generated used to specify the model settings for the second phase of analysis during which genome sizes of internal nodes were estimated using the addMRCA command. All settings were as described above for the first phase with the exception that the DataDev (DD) parameter was optimized to maximize the number of iterations within the 20–40% acceptance rate. At both stages of the analysis, parameter values were examined in tracer v1.5 to confirm that a sufficient burn‐in had been removed and stationarity reached. The final settings used for RD and DD were 0.00008 and 0.00035, respectively. Values for the ancestral genome size of subgenus *Liliorhiza* and the Eurasian clade were calculated by averaging all estimates for these nodes from the 75 001 post burn‐in iterations. The 95% confidence intervals for the mean values were calculated in R using the *t*‐test command (R Core Team, 2014). Mean and confidence interval values were back transformed in R using the following commands:


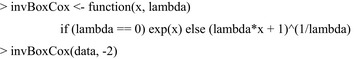


### Impact of genome size estimation method on ancestral genome size reconstruction

Because alternative genome size estimation methods (e.g. FM versus FC) can yield different results (e.g. fig. 1 in Ambrožová *et al*., 2011), we tested the impact of using 1Cx‐values from different methods on ancestral genome size reconstruction. The bayestraits analyses were repeated with *F*.* pluriflora*, the only species with a 1Cx‐value estimated by FM (Table S1), pruned from the trees (using bayestrees v1.3) and removed from the input 1Cx‐values. Parameter values were checked again to confirm that appropriate settings for RD and DD had been used (i.e. 0.00008 for RD and 0.00035 for DD), and to ensure that stationarity had been reached.

### 454 sequencing

Sequencing of total genomic DNA was conducted by the University of Liverpool Centre for Genomic Research (Liverpool, UK) and Creative Genomics (New York, NY, USA) using the Roche 454 GS FLX Titanium system; initial processing of reads, including removal of adaptor sequences, was conducted by the sequencing centres. Two runs each were performed for *F*.* affinis* and *F*.* imperialis*, generating 2428 117 and 2393 894 reads, respectively. One‐eighth of a run was generated for each of the eight remaining species, producing 86 783–118 017 reads per species. 454 sequence data have been submitted to the European Nucleotide Archive (ENA; accession no. PRJEB6757). We used cd‐hit‐454 v4.5.6 (Niu *et al*., 2010) to identify exact duplicate 454 reads (the same length with 100% similarity), which are probably artefacts of emulsion PCR (Gomez‐Alvarez *et al*., 2009), with the following parameter settings: ‐c 1 ‐aL 1 ‐aS 1 ‐D 0 ‐g 1. From clusters of duplicate reads identified by cd‐hit‐454, custom Perl scripts were used to remove redundant reads from each 454 data set (retaining a single read from each cluster).

To identify reads of organellar origin, all unique 454 reads were screened against custom databases of monocot plastid genomes (all genomes available from NCBI at the time of analysis and a draft plastid genome sequence of *Lilium superbum*; Givnish *et al*., 2010) and monocot mitochondrial genomes (all genomes available from NCBI at the time of analysis) using the stand‐alone version of Blast (v2.2.16; Altschul *et al*., 1997). Parameter settings used for Blastn searches were: ‐v 1 ‐G 0 ‐E 2 ‐K 0 ‐b 0 ‐e 0.000001 ‐F mL. Reads with a significant hit (*E*‐value  ≤ 1 × 10^−6^) to the plastid or mitochondrial databases were filtered from the 454 data sets using a custom Perl script. All remaining reads were considered to be of nuclear origin; we refer to these as the ‘unique nuclear reads’ (Table S4).

### *De novo* identification of repetitive sequence families in *Fritillaria*

To generate reference sequences for repetitive element families from the *Fritillaria* genomes, we performed graph‐based clustering of unique nuclear 454 reads using the repeatexplorer pipeline via galaxy (Novák *et al*., 2010, 2013). Clustering was performed separately for *F*.* affinis* and *F*.* imperialis* to create a reference set of repeat families for each. Initial runs of repeatexplorer revealed that the number of reads from *F*.* affinis* that it is possible to cluster is limited by the presence of a relatively high‐abundance tandem repeat (corresponding to the FriSAT1 repeat identified by Ambrožová *et al*., 2011). The number of reads that can be analysed simultaneously by repeatexplorer is governed by the number of similarity hits produced, as all read overlaps are loaded into the computer memory during the graph‐based clustering step (Novák *et al*., 2013). Consequently, this limit does not differ greatly between, for example, 200 and 400 bp reads (it is recommended that reads of the same length are used), allowing coverage to be increased by analysing longer reads. Therefore, to maximize the genome coverage for *F*.* affinis*, clustering was performed on 400 bp reads; custom Perl scripts were used to trim reads of > 400 bp from the 3′ end and to remove any reads of < 400 bp. For *F*.* affinis*, all 400 bp reads were inputted into repeatexplorer, allowing it to randomly subsample the data set to the maximum number of reads that could be processed (830 674 of 1056 953 available 400 bp reads were used). A random sample of 400 bp reads (842 670) from *F*.* imperialis* was taken using the sequence sampling tool (v1.0.0) in repeatexplorer to create a data set providing the same level of genome coverage (0.74%) as for *F*.* affinis*. The clustering pipeline was run with ≥ 220 bp overlap for clustering and ≥ 160 bp overlap for assembly. All clusters containing ≥ 0.01% of the input reads were examined manually to identify clusters that required merging (i.e. where there was evidence that a single repeat family had been split over multiple clusters). Clusters were merged if they met the following criteria: they formed connected components with a significant number of similarity hits between the clusters (e.g. in a pair of clusters, 5% of the reads in the smaller cluster had Blast hits to reads in the larger cluster); they were of the same repeat type (e.g. Copia LTR retrotransposons); they would be merged in a logical position (e.g. for repetitive elements containing conserved domains, clusters were only merged if it would result in the conserved domains being joined in the correct order). The reclustering pipeline was run using ≥ 160 bp overlap for assembly and the merged clusters were examined manually to verify that all domains were in the correct orientation.

Clusters were annotated in repeatexplorer according to hits from Blast searches to the repeatmasker Viridiplantae database and to a database of conserved domains; where a substantial number of reads matched the same repeat type (e.g. 20% of reads in the cluster matching a Gypsy LTR retrotransposon) these annotations were retained. For clusters not annotated in repeatexplorer (i.e. no significant Blast hits), or where only very few reads had a Blast hit or separate reads matched different repeat types (i.e. inconsistent Blast hits), contigs were searched against GenBank using Blastn and Blastx (Altschul *et al*., 1997) and submitted to Tandem Repeat Finder (Benson, 1999).

To calculate the proportion of the genome (genome proportion (GP)) comprised of each repeat family (i.e. cluster), we conducted Blast searches of all unique nuclear reads (Table S4) against databases of the contigs from the clustering analysis. GP was calculated for all clusters containing ≥ 0.05% of the reads inputted into repeatexplorer (Tables S5, S6; we refer to these as the ‘top’ repeat families); we used ≥ 0.05% reads as a cut‐off as these clusters contain > 165 kb of data, which is sufficient to provide several‐fold coverage for most known repetitive elements (e.g. see http://gydb.org), and therefore can be expected to represent complete elements. Contigs from all clusters were used to create separate custom Blast databases for *F*.* affinis* and *F*.* imperialis* using the makeblastdb tool in Blast+ (v2.2.24+; Camacho *et al*., 2009). The unique nuclear read data sets from each of the 10 species sequenced (Table S4) were searched against each database using megablast in the Blastn tool in Blast+ (v2.2.24+). To capture the maximum number of hits, searches were conducted with a relaxed *E*‐value of 100 and no filter for low‐complexity sequence (further increases to the *E*‐value cut‐off did not result in additional hits); a single hit was recorded for each read. Blast results were then filtered using a custom Perl script to retain only those where ≥ 55% of the query read matched one of the contigs, with ≥ 90% similarity between the query and subject in the matching portion. We calculated the GP from the filtered Blast hits using a custom Perl script. For each contig, the number of bases of the query sequence participating in the top high‐scoring pair for each Blast hit was summed to give the total number of bp representing each contig in the data sets of unique nuclear reads. For each cluster, the number of bp for all of its contigs was summed and expressed as a percentage of the total data set size (i.e. total number of bp in the set of unique nuclear reads; Table S4) to give the value for GP. The genomic abundance of each cluster in Mb was calculated as follows: (total Mb of cluster in data set × genome size in Mb/data set size in Mb). GP and Mb estimates for the top clusters in *F*.* affinis* and *F*.* imperialis* are shown in Tables S5 and S6.

### Statistical analyses

To test the relationship between the amount of single/low‐copy DNA in the genome (the S/L fraction) and overall genome size, we used data from published DNA reassociation studies (Thompson, 1978; Wenzel & Hemleben, 1982; Elsik & Williams, 2000; Table S7). Estimates of the percentage of S/L DNA (often referred to as the ‘unique’ or ‘single‐copy’ fraction in older references, but here conservatively called the S/L fraction) were used to calculate the size of this portion of the genome in Mb on the basis of the prime 1C value for each species from release 6.0 of the Plant DNA C‐values Database (Bennett & Leitch, 2012). Any duplicate values for the estimated percentage of S/L DNA (i.e. values from earlier studies compiled in later publications) were removed; where there were multiple independent estimates for a species, we averaged all the percentages and used this mean value. The size of the S/L fraction was calculated on the basis of both 1C and 1Cx genome size (Table S7). Ploidy values were taken from the Plant DNA C‐values Database, as this information was sometimes lacking in the original DNA reassociation studies (Thompson, 1978; Wenzel & Hemleben, 1982; Elsik & Williams, 2000); where the Plant DNA C‐values Database contained entries for individuals of different ploidies from the same species, we used the C values for diploids to calculate the size of the S/L fraction in Mb, as the percentage of S/L DNA estimated should be the same irrespective of ploidy. Correlation between the size of the S/L fraction per 1C and 1Cx genome and total genome size (expressed both as 1C and 1Cx‐values) was tested using Kendall's tau‐b from the ‘Kendall’ package in R for all species simultaneously and for separate plant families where data were available for at least five species (Fig. S2).

## Results

### Extreme genome size expansions occur independently in *Fritillaria*

In order to test the prediction that extreme genome size expansion occurs via the massive amplification of a few repeat families, we analysed two species of *Fritillaria* with similar monoploid genome sizes (1Cx‐value = 2C value/ploidy level; Greilhuber *et al*., 2005), *F*.* affinis* (1Cx = 44.94 Gb) and *F*.* imperialis* (1Cx = 45.59 Gb; Table S1). To verify whether genomic expansion occurred separately in these species, we reconstructed the genome size for the MRCA of each clade. The results show that both species have a genome > 9 Gb larger than the estimate for the MRCA of their clade (Fig. 1), corroborating phylogenetically independent increases in each lineage. Analyses with or without *F*.* pluriflora* (see the Materials and Methods section) yielded very similar results (35.226/35.084 Gb with/without *F*.* pluriflora* for the MRCA of the *Liliorhiza* clade; 36.192/36.162 Gb for the MRCA of the Eurasian clade). Therefore, ancestral genome size values estimated with all species were used.

### Extreme genome expansions are not governed by the activity of just a few repeat families

**Figure 2:**
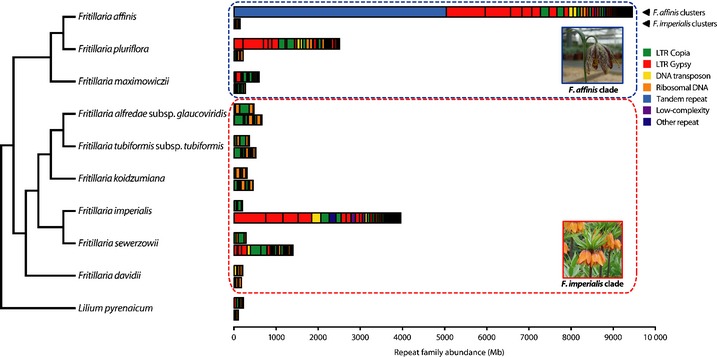
Cumulative abundance of the most common repeat families from *Fritillaria affinis* and *Fritillaria imperialis*. For each species, the abundance in their genome of the top repeat families identified from *F*.* affinis* (upper bar) and *F*.* imperialis* (lower bar) is shown in megabases (Mb). Repeat families are ordered from left to right according to their abundance in *F*.* affinis* (upper bar in each pair) and *F*.* imperialis* (lower bar in each pair) and coloured according to repeat type; LTR, long terminal repeat retrotransposon. The summary of relationships between the 10 species is derived from the phylogenetic tree shown in Fig. 1.

To identify sequences involved in these independent genome expansions, we conducted low‐pass 454 sequencing (*c*. 2% genome coverage) to capture the most highly repeated components. We clustered separately 454 reads at the same level of coverage for each species (0.74%) to identify different families of repeats. For each cluster containing ≥ 0.05% of the input reads (i.e. the ‘top’ repeat families; *n *=* *47 in *F*.* affinis* and *n *=* *41 in *F*.* imperialis*), we used all 454 reads to estimate the number of Mb and proportion of the genome (GP) comprised of this repeat (see the Materials and Methods section). The top repeats in *F*.* affinis* together account for 9.44 Gb, or 21.00% of the genome (Fig. 2; Table S5); only three individually have a GP of ≥ 1%, with the most abundant family having a GP of 11.19% (> 5 Gb; Fig. 2; Table S5). In *F*.* imperialis*, the top repeats account for 3.95 Gb, or 8.66% of the genome (Fig. 2; Table S6); the most abundant repeat has a GP of 1.64% and is the only family comprising ≥ 1% of the genome. Despite the presence in *F*.* affinis* of a repeat constituting > 11% of the genome, the top repeats together do not account fully for the estimated 9.71‐Gb expansion. In *F*.* imperialis*, amplification of the top repeats explains only 42% of the estimated expansion. Moreover, these calculations assume that the *F*.* affinis* and *F*.* imperialis* repeats were either absent in their respective ancestors or present in few copies. To test whether these repeats show evidence of specific amplification in the *F*.* affinis* and *F*.* imperialis* lineages, we estimated their abundance in eight additional species (Fig. S1). The most abundant repeats from *F*.* affinis* and *F*.* imperialis* comprise ≥ 200 and ≥ 101 Mb, respectively, in each of the nine other species (Fig. 2; Tables S5, S6). Assuming similar minimum abundances in the MRCA of their lineages, this implies that up to 9.24 Gb of the 9.44 Gb comprised of these repeats in *F*.* affinis* and up to 3.85 Gb of the 3.95 Gb in *F*.* imperialis* result from amplification subsequent to divergence from their ancestors. This accounts for 95% of the estimated expansion in *F*.* affinis* and 41% of that in *F*.* imperialis*. Thus, neither independent genome expansion fits a model of genome size increase via massive amplification of a handful of repeat families. Moreover, as these values encompass all repeat families of ≥ 0.05% GP, any remaining families have a GP of < 0.05% (equivalent to < 23 Mb per family). Consequently, *c*. 80–90% of the DNA of these species is predicted to comprise repeat families of ≤ 0.05% GP, indicating that the vast majority of their genomes are made up of a heterogeneous set of relatively low‐abundance DNA.

A potential cause of contrasting patterns of repeat diversity between species is the application of different stringency levels when delimiting repetitive element families and assessing their abundance. To test whether our approach to *de novo* identification and quantification of repeat families may be responsible for the different pattern of repeat diversity detected in *Fritillaria* to that expected on the basis of data from other species, we used the same methods to analyse data from barley (*Hordeum vulgare*), a species in which a large portion of the genome is made up of a small number of high‐abundance repeat families (Wicker *et al*., 2009; Notes S1). Results obtained by applying our approach to the analysis of data from barley agree with previous results in revealing a large fraction of the genome to be comprised of a small number of high‐abundance repeats, with the top 10 repeat families accounting for 30.33% of the genome compared with 35.38% in the analysis of Wicker *et al*. (2009). Although our approach to *de novo* repeat family identification and quantification might result in some additional families being recognized, with consequently lower abundance for individual families (Notes S1), it is clear that any difference in stringency between the methods we have used and those that have been applied elsewhere does not change the overall picture of repeat diversity in the species analysed. Consequently, the contrasting genomic composition of *F*.* affinis* and *F*.* imperialis* compared with that of other species with smaller genomes cannot be attributed simply to differences in the specific methods for characterizing repeats.

### Very large genomes show evidence for low deletion and turnover of DNA

**Figure 3:**
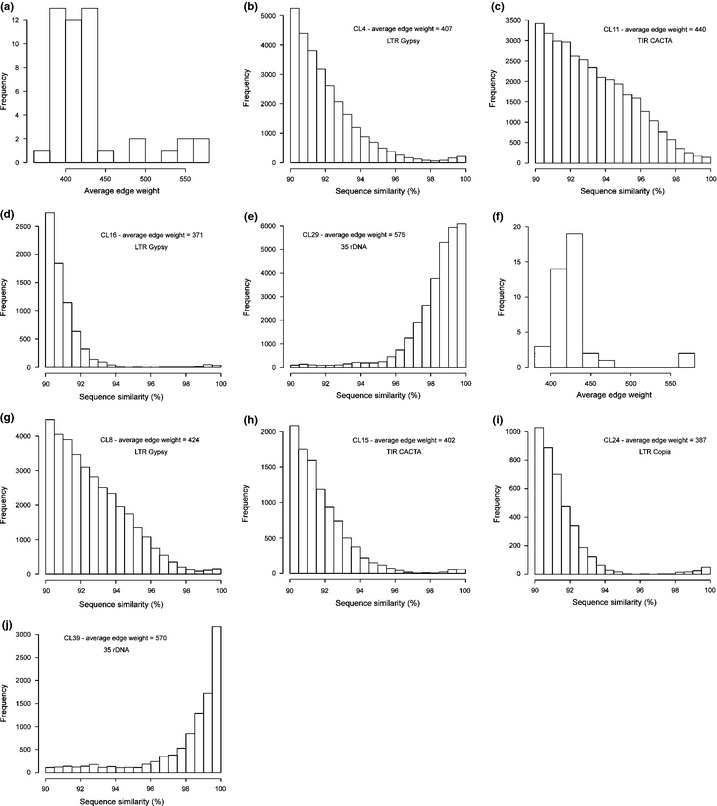
Intrafamily heterogeneity of repeats in *Fritillaria*. (a) Histogram of average edge weights from graphs of all top repeat families from *Fritillaria affinis* (*n *=* *47). (b–e) Histograms of percentage sequence similarity for read pairs from selected repeat families representing a range of different edge weights from *F. affinis*, illustrating that repeat families with average edge weights of < 450, which comprise the vast majority of the top families, show an absence of peaks of very high similarity read pairs (i.e. ≥ 98% sequence similarity). (f) Histogram of average edge weights from graphs of all top repeat families from *F. imperialis* (*n *=* *41). (g–j) Histograms of percentage sequence similarity for read pairs from selected repeat families representing a range of different edge weights from *F. imperialis*, showing a similar pattern to that described above for *F. affinis*. Cluster names and repeat types follow those listed in Supporting Information Tables S5 and S6; see Notes S2 for further explanation.

The heterogeneous repeat content in *Fritillaria* could have arisen via distinct pathways. First, global amplification of repetitive DNA and high genome turnover could result in many repeat families amplifying simultaneously but remaining relatively small in size because of rapid deletion of amplified copies. Second, simultaneous amplification of a number of different repeat families accompanied by low rates of deletion could lead amplified copies to accumulate, creating an increasing fraction of repeat‐derived DNA that degenerates and diverges over time. To distinguish between these scenarios, we examined the level of intrafamily heterogeneity for repeats in *Fritillaria* (Notes S2). This reveals that most repeat families are not made up of homogeneous copies that show evidence for recent amplification but are instead dominated by copies with relatively low similarity to each other (Fig. 3), which is consistent with a scenario of ongoing amplification and accumulation of repetitive DNA as a result of low rates of deletion.

To investigate whether other plant species also show evidence for accumulation of heterogeneous repeat‐derived DNA, we used data on the proportion of the genome comprised of single‐ or low‐copy DNA (the S/L fraction) from classical DNA reassociation studies (see the Materials and Methods section; Table S7). We find that the size of the S/L fraction is significantly positively correlated with both 1C (Kendall's tau‐b 0.784; *P *<* *2.22^−16^) and 1Cx genome size (0.816; *P *<* *2.22^−16^; Fig. S2). This relationship is also detected when analysing separately data for individual plant families (Fig. S2). Estimated sizes of the S/L fraction per monoploid genome range *c*. 70×, from 91 Mb in *Stellaria media* (Carophyllaceae) to 6338 Mb in *Anemone blanda* (Ranunculaceae; Table S7). Findings from other studies demonstrate that repeat‐derived DNA contributes to the large S/L fraction in some species and that the relationship between genome size and amount of S/L DNA cannot be explained by increases in the number of protein‐coding gene sequences (see the Discussion section).

## Discussion

By examining the repetitive DNA content of *Fritillaria* species, which have some of the largest recorded genomes in plants, we have shown that the huge size of these genomes is not determined by the activity of few high‐copy‐number TE families, as suggested to be the case in species with smaller genomes (Wicker *et al*., 2009). If species with small and large genomes differ only in their propensity for DNA amplification, with similar DNA deletion efficiency, we would expect the majority of large genomes to be made up of repetitive DNA with evidence of recent amplification, with a dearth of older, more divergent, elements. Instead, the pattern in *Fritillaria* is consistent with the accumulation and degeneration of repeat copies as a result of the failure to remove DNA as it is amplified. The approach we used for *de novo* repetitive element identification may have resulted in a slightly higher number of repeat families being inferred than would have been the case with methods used in earlier studies (see the Results section). However, the scale of the difference is not sufficient to explain the contrast between the results we obtained in *Fritillaria*, where dozens of repeat families are required to have amplified in order to explain recent genome size expansion, and the scenario proposed previously whereby very large genomes derive from massive amplification of a small number of repeat families (reviewed by Bennetzen & Wang, 2014).

Few studies have examined repetitive DNA composition in species with genomes exceeding 20 Gb (Kovach *et al*., 2010; Metcalf *et al*., 2012; Sun *et al*., 2012b), but those that have indicate the pattern of repeat diversity uncovered in *Fritillaria* may be a general characteristic of very large genomes. Only 40.2% of the *c*. 50‐Gb Australian lungfish (*Neoceratodus forsteri*) genome can be assigned to recognizable repetitive DNA (Metcalf *et al*., 2012). The black salamander (*Aneides flavipunctatus*) has a genome size of *c*. 44 Gb, < 50% of which can be assigned to known TEs (Sun *et al*., 2012b). The majority of the *c*. 22‐Gb genome of loblolly pine (*Pinus taeda*) is comprised of highly divergent, relatively low‐abundance, repetitive DNA (Kovach *et al*., 2010; Wegrzyn *et al*., 2014). In addition, the draft sequence of the Norway spruce genome (*Picea abies*), which is just below 20 Gb, reveals a similar picture (Nystedt *et al*., 2013). Although it has been suggested that the diversity of repeats in *Pinus* and *Picea* may be a specific characteristic of conifers (Kovach *et al*., 2010; Nystedt *et al*., 2013), our results demonstrate that the presence of highly heterogeneous repetitive DNA is a more widespread feature of very large genomes, a property that has also been noted recently by Metcalfe & Casane (2013).

Further support for this assertion comes from the observation that, in plants (angiosperms and gymnosperms), the amounts of both highly repeated and low‐copy DNA increase with escalating genome size (Elsik & Williams, 2000), with the amount of S/L DNA ranging from 91 Mb in *Stellaria media* (Carophyllaceae) to 6338 Mb in *Anemone blanda* (Table S7). Moreover, as these estimates encompass relatively few species, the actual range in S/L fraction size may be much greater. For example, the entire genome of *Genlisea aurea* is estimated to be *c*. 64 Mb (Greilhuber *et al*., 2006), which is smaller than the S/L fraction alone in *Stellaria media*. Repetitive sequences with 10 or more copies make up *c*. 3 Mb of the *G*.* aurea* genome assembly (Leushkin *et al*., 2013; a further 2.6 Mb of the assembly matches known repetitive elements, but the level of repetitiveness was not reported). Therefore, even if the remaining *c*. 61 Mb of the *G*.* aurea* genome is comprised solely of S/L DNA, there is a > 100‐fold difference between plant species in the amount of S/L DNA. Genome‐scale analyses have revealed the presence of multiple ancient whole‐genome duplications (WGDs) within seed plants (Jiao *et al*., 2011). Recent WGDs are accounted for when calculating the size of the S/L fraction per monoploid genome (see the Materials and Methods section; Table S7) and therefore do not inflate estimates of the size of this portion of the genome. However, ancient WGDs may not be apparent from chromosome numbers in extant species (Simillion *et al*., 2002) and, despite subsequent wide‐scale gene loss (e.g. The *Brassica rapa* Genome Sequencing Project Consortium, 2011), may contribute to increases in S/L fraction size through the retention and divergence of duplicate gene copies. Certain lineages have undergone multiple ancient WGD events; the most extensive series of duplications known are those in *Brassica* and *Gossypium*, both of which have undergone an up to 36‐fold duplication of the genes that would have been present in the MRCA of angiosperms (The *Brassica rapa* Genome Sequencing Project Consortium, 2011; Paterson *et al*., 2012). However, this still does not approach the > 100‐fold level of duplication that would be required if ancient WGDs alone were to account for variation in S/L fraction size. Furthermore, based on current understanding of the phylogenetic distribution of ancient WGDs among angiosperms (Vanneste *et al*., 2014), the largest S/L fractions are not found in lineages with the most ancient WGDs. For example, despite multiple WGDs in the *Brassica* lineage (The *Brassica rapa* Genome Sequencing Project Consortium, 2011), *Brassica rapa* subsp. *pekinensis* has a S/L fraction size of 368 Mb (Table S7), which is < 6% of the size of the S/L fraction in *A*.* blanda*. Findings such as these, added to the fact that DNA showing similarity to known TEs can be detected within the S/L sequences (Elsik & Williams, 2000; Whitelaw *et al*., 2003), suggest that it is the accumulation of repeat‐derived DNA that is primarily responsible for the large size of the S/L fraction in some species. The occurrence of escalating amounts of low‐similarity DNA with growth in overall genome size supports the conclusion that in species with large genomes repetitive DNA is retained, creating an increasing repeat‐derived fraction that decays over time to the point where its component sequences are highly divergent.

### Conclusions

Our results from *Fritillaria* demonstrate that extreme cases of genomic expansion can take place via the accumulation of highly heterogeneous, relatively low‐abundance, repeat‐derived DNA and indicate that a lack of deletion and low turnover of repetitive DNA play major roles in genome size evolution. These findings will have important consequences for understanding the content and evolution of plant genomes. Very large genomes may clearly still contain highly amplified repeat families that individually have a substantial impact on genome size, such as is shown here with the high‐abundance tandem repeat in *F. affinis* (Fig. 2). However, the overall picture we have revealed, both from analysis of genomic *Fritillaria* data and from S/L data from diverse plant species, is not one of genomes growing principally by the activity of a few repeat families as had previously been suggested. Whether very large plant genomes (> 20 Gb) exist where significant genome expansion results solely from the amplification of one or two repeat families remains to be seen. Irrespective of this, our results, as well as those from some gymnosperm and animal species, indicate that such a mode of evolution is not a general feature of extreme genome size expansions. The universality of the patterns we have revealed awaits testing with data from further species with giant genomes, such as those found in the Melanthiaceae (Pellicer *et al*., 2014) or *Viscum* (Zonneveld, 2010).

Repetitive DNA can be removed from the genome via homologous and illegitimate recombination (Fedoroff, 2012); the importance of recombination‐based processes in DNA removal is suggested by the greater estimated rate of DNA deletion in genomic regions with high recombination rates compared with those undergoing less recombination (Nam & Ellegren, 2012). A recent theory presented by Fedoroff (2012) provides a plausible mechanism by which recombination frequency, and hence DNA removal rate, might be constrained. Most repetitive elements in plant genomes are highly methylated and contained within recombinationally inert heterochromatin (Fedoroff, 2012; Henderson, 2012). It is proposed that epigenetic mechanisms, which control the formation of heterochromatin, evolved to prevent deleterious effects of unconstrained recombination (Fedoroff, 2012); if unsuppressed, the presence of multiple TE copies would be expected to stimulate large numbers of ectopic recombination events (Bennetzen & Wang, 2014). Consequently, efficient epigenetic regulation of repetitive elements may actually prevent their removal, as they become locked into tracts of the genome that cannot be accessed by the recombination machinery (Fedoroff, 2012). If this theory holds true, plant species with large genomes may accumulate more repetitive DNA because of the rapid action of epigenetic mechanisms subsequent to amplification, whereas epigenetic silencing is predicted to reach completion more slowly in species with smaller genomes, providing a window of opportunity for removal of repetitive DNA via recombination before heterochromatinization is achieved. This argument runs counter to the suggestion that epigenetic silencing of repetitive DNA may be less effective in species with large genomes, thus allowing TEs to proliferate more easily (Kelly & Leitch, 2011). Although epigenetic mechanisms involved in regulating activity of repetitive elements have been examined in limited taxa, there is evidence that they may be less efficient in the larger genome of *Arabidopsis lyrata* (1C = 245 Mb; Lysak *et al*., 2009) than in the smaller genome of *A*.* thaliana* (Hollister *et al*., 2011). However, initial evidence on the function of epigenetic mechanisms in *F. imperialis* indicates that this species shows all the signatures that are usually associated with strict epigenetic regulation of repetitive DNA in small genomes (Becher *et al*., 2014). Nevertheless, irrespective of whether greater efficiency of epigenetic control has a role in stimulating genome size expansion, our results provide clear evidence that a key factor in the evolution of very large genomes is a lack of DNA removal leading to ongoing accumulation and low turnover of repetitive and repeat‐derived sequences.

## Supporting information

Please note: Wiley Blackwell are not responsible for the content or functionality of any supporting information supplied by the authors. Any queries (other than missing material) should be directed to the *New Phytologist* Central Office.

**Fig. S1** Phylogenetic relationships between *Fritillaria* species.
**Fig. S2** Relationship between single/low‐copy sequence fraction size and genome size.
**Table S1** Monoploid genome sizes used in ancestral state reconstruction
**Table S2** Plant material used for sequencing and genome size estimation
**Table S3** Newly generated 1C values
**Table S4** Summary of 454 sequence data obtained for each species
**Table S5** Top repeat families from *Fritillaria affinis*
**Table S6** Top repeat families from *Fritillaria imperialis*
**Table S7** Single/low‐copy fraction size and genome size
**Notes S1** Potential impact of sequence similarity thresholds on repeat diversity patterns.
**Notes S2** Analysis of intrafamily heterogeneity of repeats in *Fritillaria*.
