# Supplementary material for: Analysis of the giant genomes of Fritillaria (Liliaceae) indicates that a lack of DNA removal characterizes extreme expansions in genome size
Source: New Phytol. 2015 Jun 8;208(2):596–607. doi: 10.1111/nph.13471 (PMC4744688; doi:10.1111/nph.13471)
Supplement: Supplementary file 1 — Fig. S1 Phylogenetic relationships between Fritillaria species. Fig. S2 Relationship between single/low‐copy sequence fraction size and genome size. Table S1 Monoploid genome sizes used in ancestral state reconstruction Table S2 Plant material used for sequencing and genome size estimation Table S3 Newly generated 1C values Table S4 Summary of 454 sequence data obtained for each species Table S5 Top repeat families from Fritillaria affinis Table S6 Top repeat families from Fritillaria imperialis Table S7 Single/low‐copy fraction size and genome size Notes S1 Potential impact of sequence similarity thresholds on repeat diversity patterns. Notes S2 Analysis of intrafamily heterogeneity of repeats in Fritillaria. [file NPH-208-596-s001.pdf]

New Phytologist **Supporting Information Figs S1 & S2, Tables S1-S7 and Notes S1 & S2**

Article title: Analysis of the giant genomes of *Fritillaria* (Liliaceae) indicates that a lack of DNA removal characterizes extreme expansions in genome size.

Authors: Laura J. Kelly, Simon Renny-Byfield, Jaume Pellicer, Jiří Macas, Petr Novák, Pavel Neumann, Martin A. Lysak, Peter D. Day, Madeleine Berger, Michael F. Fay, Richard A. Nichols, Andrew R. Leitch and Ilia J. Leitch.

The following Supporting Information is available for this article:

Fig. S1 Phylogenetic relationships between *Fritillaria affinis*, *F. imperialis* and related species.

Fig. S2 Relationship between the size of the single/low-copy (S/L) sequence fraction and genome size.

Table S1 Monoploid genome sizes used in ancestral state reconstruction.

Table S2 Plant material used for sequencing and genome size estimation.

Table S3 Newly generated 1C-values.

Table S4 Summary of 454 sequence data obtained for each species after filtering for duplicate and organellar reads.

Table S5 Top repeat families from *Fritillaria affinis*.

Table S6 Top repeat families from *Fritillaria imperialis*.

Table S7 Single/low-copy fraction size and genome size.

Notes S1 Potential impact of differing sequence similarity thresholds on patterns of repeat diversity.

Notes S2 Analysis of intra-family heterogeneity of repeats in *Fritillaria*.

Supplementary References

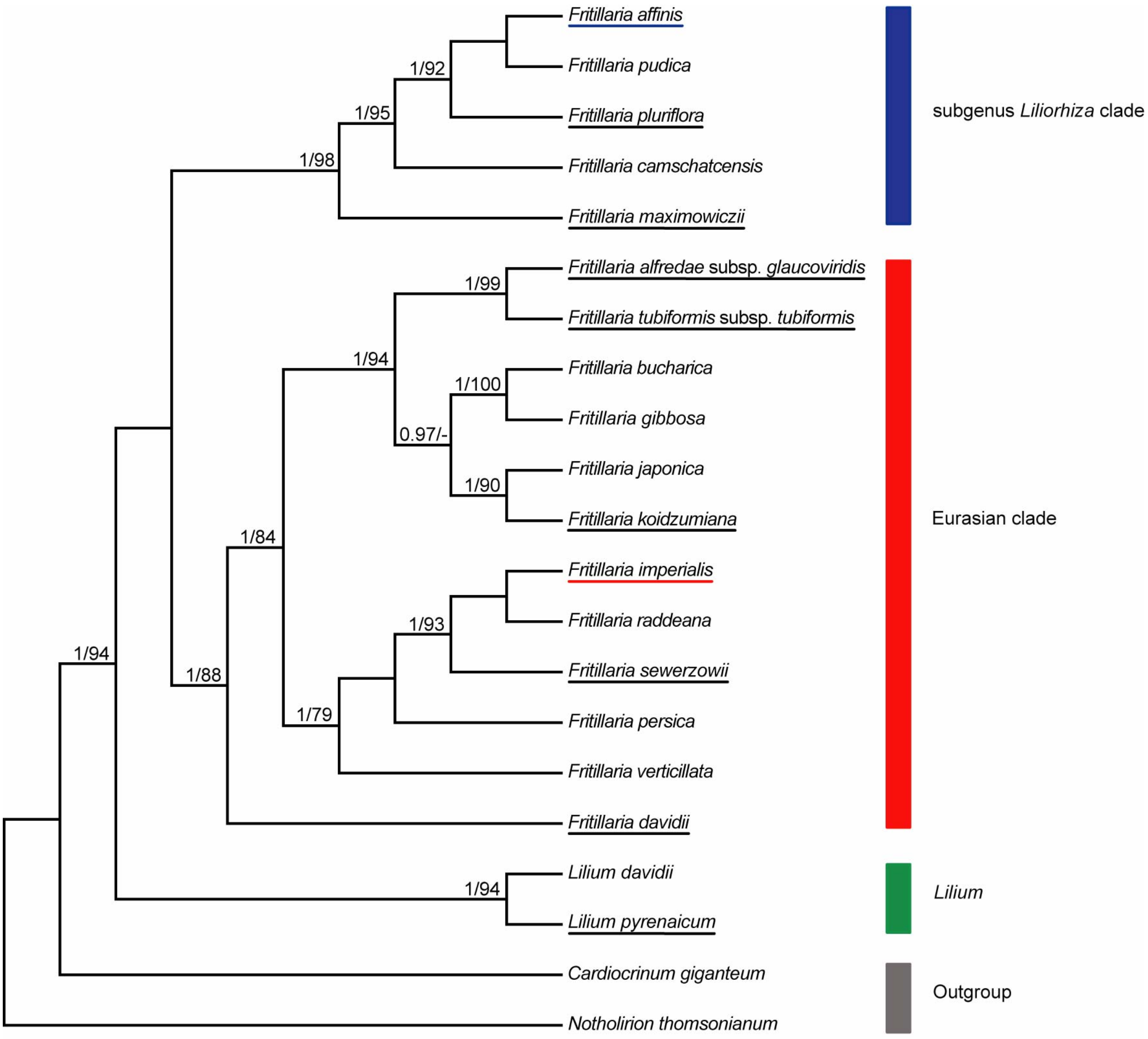

Fig. S1 Phylogenetic relationships between *Fritillaria affinis* and *F. imperialis* (the key taxa analysed in this study) and related species. Majority rule consensus tree with all compatible groupings, from the Bayesian analysis. Values above branches indicate node support (posterior probabilities (PP) of ≥ 0.95/bootstrap percentages (BP) ≥ 70); a dash indicates a node with PP ≥ 0.95 but BP < 70. PP values of < 0.95 and BP values of < 70 are not shown. The two major groups of species within *Fritillaria* are indicated: the subgenus *Liliorhiza* clade is comprised only of members of this subgenus (including *F. affinis*, underlined in blue), which occur mainly in North America; the Eurasian clade contains members of all other subgenera of *Fritillaria* (including *F. imperialis*, underlined in red), encompassing species from Europe, North Africa, the Middle East, Central Asia and China. Names underlined in black indicate species subjected to low-pass 454 sequencing in addition to *F. affinis* and *F. imperialis*.

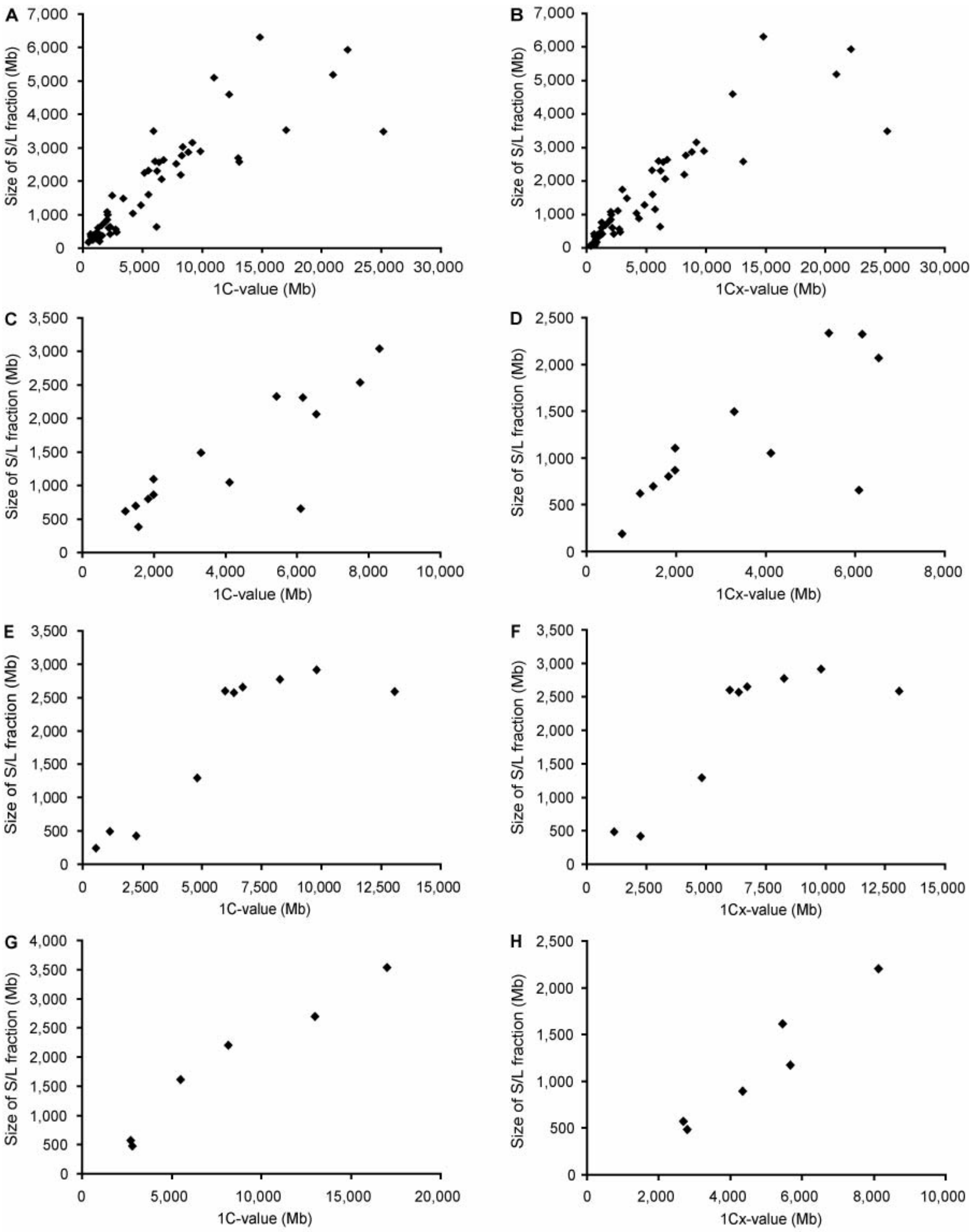

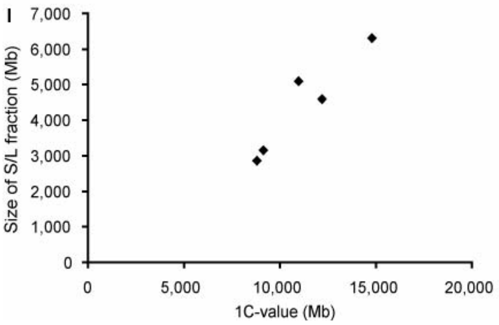

J

	Size of S/L fraction versus 1C-values		Size of S/L fraction versus 1Cx-values	
	Tau-b	*P*	Tau-b	*P*
All species (*n* = 57/52)*	0.784	< 2.22e-16	0.816	< 2.22e-16
Asteraceae (*n* = 14/12)	0.685	0.000824	0.626	0.005971
Fabaceae (*n* = 10/9)	0.733	0.004208	0.667	0.016489
Poaceae (*n* = 6)	0.867	0.024171	0.733	0.060289
Ranunculaceae (*n* = 5)	0.800	0.086411	n/a	n/a

Fig. S2 Relationship between the size of the single/low-copy (S/L) sequence fraction and genome size. (a) Scatter plot showing S/L fraction size versus 1C genome size, including data from all species (n = 57). (b) Scatter plot showing S/L fraction size versus 1Cx genome size, including data from all species (n = 52). (c) Scatter plot showing S/L fraction size versus 1C genome size, including data from Asteraceae (n = 14). (d) Scatter plot showing S/L fraction size versus 1Cx genome size, including data from Asteraceae (n = 12). (e) Scatter plot showing S/L fraction size versus 1C genome size, including data from Fabaceae (n = 10). (f) Scatter plot showing S/L fraction size versus 1Cx genome size, including data from Fabaceae (n = 9). (g) Scatter plot showing S/L fraction size versus 1C genome size, including data from Poaceae (n = 6). (h) Scatter plot showing S/L fraction size versus 1Cx genome size, including data from Poaceae (n = 6). (i) Scatter plot showing S/L fraction size versus 1C genome size, including data from Ranunculaceae (n = 5). (j) Results of correlation tests (Kendall's tau-b) between S/L fraction size and genome size (*fewer species are included for the tests with 1Cx genome size because ploidy information was not available for all taxa; correlation between S/L fraction size and 1Cx genome size was not tested for in Ranunculaceae because there were < 5 species with ploidy data). Data used to construct these plots are included in Table S7.

Table S1 Monoploid genome sizes used in ancestral state reconstruction.

Species	1Cx-value* (Gb)	Reference
Cardiocrinum giganteum	38.533	This study
Fritillaria affinis	44.939	This study
Fritillaria alfredae subsp. *glaucoviridis*	63.785	This study
Fritillaria bucharica	44.118	This study
Fritillaria camschatcensis	37.555	Ambrožová *et al.,* (2011)
Fritillaria davidii	33.252	This study
Fritillaria gibbosa	41.819	This study
Fritillaria imperialis	45.588	This study
Fritillaria japonica	85.379	Ambrožová *et al.,* (2011)
Fritillaria koidzumiana	85.242	This study
Fritillaria maximowiczii	33.536	This study
Fritillaria persica	40.124	This study
Fritillaria pluriflora	40.616	Hanson et al.†
Fritillaria pudica	37.457	Ambrožová *et al.,* (2011)
Fritillaria raddeana	41.643	This study
Fritillaria sewerzowii	43.472	This study
Fritillaria tubiformis subsp. *tubiformis*	44.010	This study
Fritillaria verticillata	40.724	This study
Lilium davidii	38.005	This study
Lilium pyrenaicum	37.976	This study
Notholirion thomsonianum	36.607	This study

*1Cx-values (monoploid genome size, c.f. Greilhuber *et al.,* 2005) were calculated by dividing the 2C-value by ploidy (see Table S3).
†Value listed in Plant DNA C-values Database (source - Hanson L, Leitch IJ, Bennett MD. Jodrell Laboratory, Royal Botanic Gardens, Kew); material from Kew Living Collection 2004-3476 was measured using Feulgen microdensitometry as described in Hanson *et al.,* (2001). Material inferred as diploid on basis of its 1C-value being close to that for diploid material from its close relative *F. affinis* (see Fig. S1 and Table S3).

Table S2 Plant material used for sequencing and genome size estimation.

Species	Collection accession[*]	DNA bank number[†]	Voucher details[‡]	454	Sanger[§]	Flow cytometry
Cardiocrinum giganteum	KLC 1988-4907	3689	Chase 3689; K	—	X	X
Fritillaria affinis	KLC 2010-905	33601	Chase 31485; K	X	X	X
Fritillaria alfredae subsp. *glaucoviridis*	LH 744	37858	Fritillaria Icones 744	X	X	X
Fritillaria bucharica	LH 488	37861	Fritillaria Icones 488	—	X	—
Fritillaria bucharica	KLC 2010-917	n/a	Photo	—	—	X
Fritillaria camschatcensis	LH 617	31539	Fritillaria Icones 617	—	X	—
Fritillaria davidii	KLC 2004-3461	25690	n/a	X	X	—
Fritillaria davidii	KLC 1992-3705	n/a	n/a	—	—	X
Fritillaria gibbosa	KLC 2004-3469	31559	Chase 31559; K	—	X	X
Fritillaria imperialis	KLC s.n.	33597	n/a	X	X	—
Fritillaria imperialis	1973-19742; KLC s.n.[¶]	n/a	Photo	—	—	X
Fritillaria japonica	LH 323	31543	n/a	—	X	—
Fritillaria koidzumiana	KLC 1979-1888	31496/37750	1983; K[‖]	X	X	X
Fritillaria maximowiczii	KLC 2005-2043	33600	Chase 31497; K	X	X	X
Fritillaria persica	KLC 1923-41201	3496	Chase 3496; K	—	X	—
Fritillaria persica	KLC 2010-1774, KLC s.n.**	n/a	Photo	—	—	X
Fritillaria pluriflora	LH 084	37775	Fritillaria Icones 084	X	X	—
Fritillaria pudica	KLC 1986-6110	24359	Photo	—	X	—
Fritillaria raddeana	KLC 1973-54	745	Chase 745; K	—	X	—
Fritillaria raddeana	KLC 1966-65810	n/a	Photo	—	—	X
Fritillaria sewerzowii	KLC 1995-4397	37751	Photo	X	X	X
Fritillaria tubiformis subsp. *tubiformis*	KLC 1966-109	2558/24360	Chase 2558; K	X	X	X
Fritillaria verticillata	KLC 2005-2049	24363	Photo	—	X	X
Lilium davidii	KLC 1979-867	3697	Chase 3697; K	—	X	X
Lilium pyrenaicum	KLC 1995-1667	37918	Chase 8639; K	X	X	X
Notholirion thomsonianum	KLC 1970-4025	448	Chase 448; K	—	X	X

[*]KLC – Kew living collection; accession numbers for material cultivated at the Royal Botanic Gardens, Kew. LH – Laurence Hill; accession numbers for material cultivated by Laurence Hill, Petersham Lodge (www.fritillariaicones.com). s.n. – without accession number.
[†]Accession numbers for the DNA Bank at the Royal Botanic Gardens, Kew (http://data.kew.org/dnabank/homepage.html). Where two numbers are listed the first extraction was used for Sanger sequencing and the second for 454 sequencing.
[‡]K – The Herbarium at the Royal Botanic Gardens, Kew. Accessions from Laurence Hill have photographic vouchers (Fritillaria Icones), which can be accessed as PDFs online at: www.fritillariaicones.com/icones/Icones.html. Accessions marked 'photo' have available photographs of the plant in flower; these are available on request from L.J.K. (l.kelly@qmul.ac.uk). Accessions marked "n/a" do not have a voucher specimen.
[§]Sanger sequences for *Fritillaria davidii*, *F. imperialis, F. japonica* and *F. koidzumiana* were newly generated; GenBank accession numbers: KP998197 - KP998208. All other sequences were taken from Day *et al.,* (2014); see Table S4 in Day *et al.,* (2014) for accession numbers.
[¶] For *F. imperialis*, fresh leaf material for the same plant as used for sequencing was not available for genome size estimation, and instead five alternative plants (including four without accession numbers) were used.
[||] Same material as Laurence Hill accession 485; photographic voucher available at: www.fritillariaicones.com/icones/ic400/Fritillaria_Icones485.pdf
[**] For *F. persica*, fresh leaf material for the same plant as used for sequencing was not available for genome size estimation, and instead three alternative plants (including two without accession numbers) were used.

Table S3 Newly generated 1C-values.

Species	1C (pg, mean ± s.d.)	1C (Mb)[*]	Ploidy[†]
Cardiocrinum giganteum	39.40 ± 0.22	38,533	2×[‡]
Fritillaria affinis	45.95 ± 0.59	44,939	2×
Fritillaria alfredae subsp. *glaucoviridis*	65.22 ± 0.48	63,785	2×[§]
Fritillaria bucharica	45.11 ± 0.19	44,118	2×
Fritillaria davidii	34.00 ± 0.35	33,252	2×[‡]
Fritillaria gibbosa	42.76 ± 0.35	41,819	2×[‡]
Fritillaria imperialis	46.01 ± 0.17	44,998	2×[¶]
Fritillaria imperialis	46.61 ± 0.10	45,585	2×[¶]
Fritillaria imperialis	46.62 ± 0.11	45,594	2×
Fritillaria imperialis	46.82 ± 0.11	45,790	2×
Fritillaria imperialis	47.01 ± 0.21	45,976	2×
Fritillaria koidzumiana	87.16 ± 0.26	85,242	2×
Fritillaria maximowiczii	34.29 ± 0.06	33,536	2×[‖]
Fritillaria persica	40.65 ± 0.37	39,756	2×[‖]
Fritillaria persica	41.06 ± 0.18	40,157	2×[‖]
Fritillaria persica	41.37 ± 0.13	40,460	2×[‖]
Fritillaria raddeana	42.58 ± 0.09	41,643	2×[‖]
Fritillaria sewerzowii	44.45 ± 0.40	43,472	2×
Fritillaria tubiformis subsp. *tubiformis*	45.00 ± 0.19	44,010	2×[‡]
Fritillaria verticillata	41.64 ± 0.13	40,724	2×[‖]
Lilium davidii	38.86 ± 0.38	38,005	2×[**]
Lilium pyrenaicum	38.83 ± 0.09	37,976	2×[**]
Notholirion thomsonianum	37.43 ± 0.02	36,607	2×[‡]

[*]1 pg = 978 Mbp (Doležel et al 2003).
[†]Unless otherwise indicated, ploidy was verified on the basis of chromosome counts carried out on the same plant as used for genome-size estimation.
[‡]Inferred from published chromosome count for the same living accession from Leitch *et al.,* (2007) or Ambrožová et al (2011).
[§]Material inferred as diploid on basis of its 1C-value being close to that for the diploid *F. alfredae* subsp. *glaucoviridis* accession measured in Leitch *et al.,* (2007).
[¶]Material inferred as diploid on basis of its 1C-value being close to that for the other *F. imperialis* accessions where chromosome counts were made.
[‖]Material inferred as diploid on basis of its 1C-value being close to that for the diploid accessions of the same species measured in Leitch *et al.,* (2007), Ambrožová et al (2011) or Fujimoto *et al.,* (2005).

[**]Material inferred as diploid on basis of 1C-values being close to those for diploid individuals of these species measured previously (see Plant DNA C-values database release 6.0, http://data.kew.org/cvalues/).

Table S4 Summary of 454 sequence data obtained for each species after filtering for duplicate and organellar reads.

**Species***	**Number of reads**	**Total Mb**	**Genome coverage† (%)**
A			
Fritillaria affinis	2,348,745	821.58	1.83
Fritillaria imperialis	2,274,576	816.48	1.79
B			
Fritillaria alfredae subsp. *glaucoviridis*	98,843	28.11	0.04
Fritillaria davidii	114,387	36.60	0.11
Fritillaria koidzumiana	80,685	29.23	0.03
Fritillaria maximowiczii	89,997	33.20	0.10
Fritillaria pluriflora	105,790	37.69	0.09
Fritillaria sewerzowii	95,794	33.99	0.08
Fritillaria tubiformis subsp. *tubiformis*	87,315	33.25	0.08
Lilium pyrenaicum	103,035	30.55	0.08

*Set A – two plates of 454 sequencing performed per species; set B – one lane of 454 sequencing performed per species (see Materials and Methods).
†Based on genome sizes listed in Table S1.

Table S5 Top repeat families from *Fritillaria affinis*.

Rank[*]	Name[†]	Repeat Type	Estimated abundance (Mb)/proportion of the genome (%)[‡]									
			Fritillaria affinis	*Fritillaria alfredae* subsp. *glaucoviridis*	*Fritillaria davidii*	*Fritillaria imperialis*	*Fritillaria koidzumiana*	*Fritillaria maximowiczii*	*Fritillaria pluriflora*	*Fritillaria sewerzowii*	*Fritillaria tubiformis* subsp. *tubiformis*	*Lilium pyrenaicum*
1	CL1	Tandem repeat	5029.14/11.19	0.00/0.00	0.00/0.00	0.04/0.00	0.00/0.00	0.00/0.00	4.18/0.01	0.00/0.00	0.00/0.00	0.05/0.00
2	CL2	LTR: Gypsy	922.58/2.05	0.00/0.00	0.06/0.00	0.02/0.00	0.29/0.00	11.23/0.03	208.28/0.51	0.00/0.00	0.00/0.00	52.43/0.14
3	CL3	LTR: Gypsy	597.33/1.33	0.13/0.00	0.11/0.00	0.12/0.00	0.00/0.00	19.44/0.06	486.62/1.20	0.22/0.00	0.06/0.00	0.14/0.00
4	CL4	LTR: Gypsy	268.05/0.60	0.00/0.00	0.00/0.00	0.01/0.00	0.00/0.00	18.51/0.06	108.58/0.27	0.00/0.00	0.00/0.00	0.00/0.00
5	CL5	LTR: Gypsy	233.670.52/	0.12/0.00	0.10/0.00	0.02/0.00	0.24/0.00	0.00/0.00	86.18/0.21	0.00/0.00	0.06/0.00	0.00/0.00
6	CL8	LTR: Gypsy	206.80/0.46	0.00/0.00	0.99/0.00	0.44/0.00	0.37/0.00	113.93/0.34	164.92/0.41	0.11/0.00	0.00/0.00	0.00/0.00
7	CL6	LTR: Copia	203.98/0.45	1.75/0.00	3.59/0.01	1.21/0.00	0.00/0.00	3.90/0.01	155.11/0.38	0.30/0.00	1.60/0.00	0.27/0.00
8	CL7	LTR: Gypsy	183.99/0.41	0.00/0.00	0.00/0.00	0.04/0.00	0.00/0.00	2.15/0.01	39.43/0.10	0.00/0.00	0.00/0.00	0.00/0.00
9	CL9	LTR: Copia	170.79/0.38	0.00/0.00	0.00/0.00	0.00/0.00	0.00/0.00	0.00/0.00	185.60/0.46	0.00/0.00	0.00/0.00	56.18/0.15
10	CL10	LTR: Gypsy	108.270.24/	0.00/0.00	0.00/0.00	0.01/0.00	0.00/0.00	0.00/0.00	51.45/0.13	0.00/0.00	0.00/0.00	0.00/0.00
11	CL11	TIR: CACTA	107.94/0.24	0.13/0.00	1.19/0.00	0.33/0.00	0.00/0.00	24.89/0.07	82.39/0.20	0.07/0.00	0.56/0.00	0.06/0.00
12	CL12	TIR: CACTA	90.77/0.20	5.05/0.01	59.17/0.18	5.95/0.01	3.76/0.00	22.09/0.07	54.52/0.13	4.48/0.01	14.94/0.03	0.47/0.00
13	CL13	LTR: Copia	75.07/0.17	22.27/0.03	15.13/0.05	50.18/0.11	40.38/0.05	85.11/0.25	78.30/0.19	49.02/0.11	51.39/0.12	2.89/0.01
14	CL14	5S rDNA	74.86/0.17	2.12/0.00	13.81/0.04	0.08/0.00	1.93/0.00	29.26/0.09	86.31/0.21	0.00/0.00	0.00/0.00	0.00/0.00
15	CL18	35S rDNA	67.48/0.15	96.62/0.15	46.49/0.14	26.94/0.06	94.90/0.11	27.63/0.08	73.30/0.18	49.10/0.11	64.06/0.15	29.87/0.08
16	CL16	LTR: Gypsy	64.81/0.14	0.00/0.00	0.00/0.00	0.00/0.00	0.00/0.00	0.00/0.00	12.81/0.03	0.00/0.00	0.00/0.00	0.00/0.00
17	CL15	LTR: Copia	64.47/0.14	0.52/0.00	0.46/0.00	0.38/0.00	0.00/0.00	72.34/0.22	9.47/0.02	0.44/0.00	0.00/0.00	0.00/0.00
18	CL19	LTR: Gypsy	61.51/0.14	4.11/0.01	0.00/0.00	0.00/0.00	1.19/0.00	18.42/0.05	63.77/0.16	0.00/0.00	1.01/0.00	0.00/0.00
19	CL20	Low complexity	59.69/0.13	0.00/0.00	0.00/0.00	0.00/0.00	0.00/0.00	0.32/0.00	5.33/0.01	0.00/0.00	0.00/0.00	0.00/0.00
20	CL17	LTR: Gypsy	58.43/0.13	0.00/0.00	0.00/0.00	0.00/0.00	0.00/0.00	0.00/0.00	29.28/0.07	0.00/0.00	0.00/0.00	0.16/0.00
21	CL21	LTR: Copia	54.29/0.12	0.00/0.00	0.00/0.00	0.97/0.00	22.06/0.03	10.10/0.03	8.66/0.02	0.68/0.00	4.55/0.01	0.00/0.00
22	CL22	LTR: Copia	52.43/0.12	222.62/0.35	0.00/0.00	84.24/0.18	39.93/0.05	37.86/0.11	71.99/0.18	137.18/0.32	139.41/0.32	0.00/0.00

23	CL27	Tandem repeat	42.23/0.09	0.00/0.00	0.00/0.00	0.25/0.00	0.00/0.00	2.38/0.01	2.41/0.01	0.00/0.00	0.00/0.00	0.00/0.00
24	CL24	LTR: Gypsy	40.01/0.09	0.10/0.00	0.00/0.00	0.01/0.00	0.00/0.00	4.72/0.01	16.37/0.04	0.00/0.00	0.00/0.00	0.00/0.00
25	CL29	35S rDNA	37.99/0.08	91.30/0.14	56.05/0.17	20.71/0.05	77.18/0.09	35.17/0.10	59.17/0.15	44.68/0.10	55.89/0.13	37.09/0.10
26	CL23	LTR: Gypsy	37.77/0.08	0.00/0.00	0.00/0.00	0.03/0.00	0.00/0.00	0.00/0.00	20.61/0.05	0.00/0.00	0.00/0.00	0.00/0.00
27	CL30	LTR: Gypsy	37.39/0.08	0.00/0.00	0.00/0.00	0.02/0.00	0.00/0.00	3.00/0.01	22.33/0.05	0.00/0.00	0.22/0.00	0.00/0.00
28	CL25	LTR: Gypsy	35.55/0.08	0.00/0.00	0.00/0.00	0.01/0.00	0.00/0.00	0.00/0.00	12.16/0.03	0.00/0.00	0.00/0.00	0.00/0.00
29	CL28	LTR: Copia	33.44/0.07	0.00/0.00	0.00/0.00	0.00/0.00	0.00/0.00	0.00/0.00	22.94/0.06	0.00/0.00	0.00/0.00	2.08/0.01
30	CL26	LTR: Gypsy	32.64/0.07	0.00/0.00	0.00/0.00	0.05/0.00	0.00/0.00	0.11/0.00	16.00/0.04	0.00/0.00	0.00/0.00	0.00/0.00
31	CL31	LTR: Gypsy	31.06/0.07	0.37/0.00	10.28/0.03	2.25/0.00	0.00/0.00	16.87/0.05	29.57/0.07	0.37/0.00	4.71/0.01	0.33/0.00
32	CL32	LTR: Copia	29.45/0.07	9.17/0.01	0.00/0.00	0.92/0.00	14.45/0.02	2.35/0.01	29.89/0.07	0.52/0.00	10.06/0.02	25.63/0.07
33	CL34	Low complexity	27.92/0.06	0.00/0.00	0.00/0.00	0.01/0.00	0.00/0.00	1.17/0.00	12.95/0.03	0.00/0.00	0.00/0.00	0.00/0.00
34	CL33	LTR: Gypsy	26.24/0.06	0.00/0.00	0.00/0.00	0.03/0.00	0.00/0.00	5.92/0.02	52.86/0.13	0.00/0.00	0.00/0.00	0.00/0.00
35	CL35	Low complexity	25.92/0.06	0.00/0.00	0.00/0.00	0.00/0.00	0.00/0.00	0.00/0.00	10.94/0.03	0.00/0.00	0.00/0.00	0.00/0.00
36	CL40	5S rDNA	24.49/0.05	11.45/0.02	0.67/0.00	0.95/0.00	10.69/0.01	4.39/0.01	3.87/0.01	0.29/0.00	3.38/0.01	4.09/0.01
37	CL39	LTR: Copia	23.88/0.05	0.00/0.00	0.00/0.00	0.00/0.00	0.00/0.00	0.00/0.00	12.33/0.03	0.00/0.00	0.00/0.00	1.35/0.00
38	CL37	LTR: Gypsy	23.79/0.05	4.05/0.01	0.67/0.00	1.81/0.00	1.86/0.00	8.17/0.02	43.18/0.11	1.73/0.00	7.48/0.02	1.49/0.00
39	CL42	Helitron	21.39/0.05	0.00/0.00	0.00/0.00	0.03/0.00	0.00/0.00	4.87/0.01	9.81/0.02	0.00/0.00	0.00/0.00	0.00/0.00
40	CL41	LTR: Copia	20.53/0.05	0.00/0.00	0.00/0.00	1.76/0.00	2.82/0.00	3.13/0.01	18.69/0.05	2.18/0.01	1.06/0.00	1.41/0.00
41	CL36	Low complexity	20.34/0.05	0.00/0.00	0.00/0.00	0.00/0.00	0.00/0.00	0.00/0.00	0.51/0.00	0.00/0.00	0.00/0.00	0.00/0.00
42	CL46	LTR: Gypsy	19.73/0.04	0.00/0.00	0.00/0.00	0.00/0.00	0.00/0.00	0.00/0.00	10.18/0.03	0.00/0.00	0.00/0.00	0.00/0.00
43	CL43	LTR: Copia	19.21/0.04	0.00/0.00	0.00/0.00	0.00/0.00	0.00/0.00	0.00/0.00	10.15/0.02	0.00/0.00	0.00/0.00	2.94/0.01
44	CL38	LTR: Gypsy	19.20/0.04	0.00/0.00	0.00/0.00	0.00/0.00	0.00/0.00	0.00/0.00	5.58/0.01	0.00/0.00	0.00/0.00	0.00/0.00
45	CL47	LTR: Gypsy	18.86/0.04	0.00/0.00	0.00/0.00	0.05/0.00	0.00/0.00	0.00/0.00	11.25/0.03	0.00/0.00	0.00/0.00	0.00/0.00
46	CL45	Low complexity	15.67/0.03	0.00/0.00	0.00/0.00	0.00/0.00	0.00/0.00	0.00/0.00	1.86/0.00	0.00/0.00	0.00/0.00	0.00/0.00
47	CL44	Low complexity	14.41/0.03	0.00/0.00	0.00/0.00	0.00/0.00	0.00/0.00	0.00/0.00	2.04/0.01	0.00/0.00	0.00/0.00	0.00/0.00
**TOTAL**			9435.43/21.00	471.88/0.74	208.76/0.63	199.87/0.44	312.06/0.37	589.42/1.76	2504.13/6.17	291.35/0.67	360.44/0.82	218.94/0.58

[*]Clusters are ranked in order of their abundance in *F. affinis*.

[†]Names from RepeatExplorer.

[‡]Given to 2 dp. Values for *F. affinis* are shown first; other species are then listed alphabetically.

Table S6 Top repeat families from *Fritillaria imperialis*.

Rank[*]	Name[†]	Repeat Type	Estimated abundance (Mb)/proportion of the genome (%)[‡]									
			Fritillaria imperialis	***Fritillaria affinis***	***Fritillaria alfredae* subsp. *glaucoviridis***	***Fritillaria davidii***	***Fritillaria koidzumiana***	***Fritillaria maximowiczii***	***Fritillaria pluriflora***	***Fritillaria sewerzowii***	***Fritillaria tubiformis* subsp. *tubiformis***	***Lilium pyrenaicum***
1	CL1	LTR: Gypsy	749.25/1.64	0.34/0.00	0.12/0.00	0.39/0.00	0.85/0.00	0.16/0.00	0.06/0.00	73.14/0.17	0.66/0.00	0.09/0.00
2	CL2	LTR: Gypsy	406.04/0.89	0.08/0.00	0.36/0.00	0.00/0.00	0.27/0.00	0.03/0.00	0.06/0.00	24.56/0.06	0.00/0.00	0.00/0.00
3	CL3	LTR: Gypsy	358.28/0.79	0.14/0.00	0.00/0.00	0.00/0.00	0.00/0.00	0.00/0.00	0.23/0.00	81.15/0.19	0.00/0.00	0.00/0.00
4	CL4	LTR: Gypsy	325.07/0.71	0.07/0.00	1.24/0.00	0.00/0.00	4.30/0.01	0.08/0.00	0.00/0.00	128.15/0.29	0.27/0.00	0.00/0.00
5	CL5	TIR: CACTA	213.91/0.47	2.89/0.01	7.73/0.01	13.36/0.04	1.55/0.00	1.33/0.00	1.03/0.00	82.17/0.19	5.35/0.01	0.40/0.00
6	CL6	LTR: Copia	202.28/0.44	26.47/0.06	212.00/0.33	0.00/0.00	74.87/0.09	34.36/0.10	39.96/0.01	244.82/0.56	157.22/0.36	0.00/0.00
7	CL7	Pararetrovirus	159.72/0.35	0.03/0.00	0.00/0.00	0.00/0.00	0.00/0.00	0.00/0.00	0.00/0.00	0.00/0.00	0.00/0.00	0.00/0.00
8	CL9	LTR: Copia	122.89/0.27	8.48/0.02	27.85/0.04	0.00/0.00	0.00/0.00	25.46/0.08	7.61/0.00	143.94/0.33	31.36/0.07	0.00/0.00
9	CL8	LTR: Gypsy	121.12/0.27	0.05/0.00	2.00/0.00	0.05/0.00	0.00/0.00	0.12/0.00	0.04/0.00	73.11/0.17	0.87/0.00	0.00/0.00
10	CL10	LTR: Gypsy	111.60/0.24	0.06/0.00	0.00/0.00	0.00/0.00	0.00/0.00	0.00/0.00	0.18/0.00	18.91/0.04	0.00/0.00	0.00/0.00
11	CL11	Low complexity	106.06/0.23	0.03/0.00	0.00/0.00	0.00/0.00	0.00/0.00	0.00/0.00	0.21/0.00	8.36/0.02	0.00/0.00	0.00/0.00
12	CL12	LTR: Gypsy	94.68/0.21	0.06/0.00	0.20/0.00	0.05/0.00	0.00/0.00	0.00/0.00	0.10/0.00	28.30/0.07	0.43/0.00	0.13/0.00
13	CL14	LTR: Gypsy	65.94/0.14	0.00/0.00	0.00/0.00	0.06/0.00	0.00/0.00	0.00/0.00	0.00/0.00	0.00/0.00	0.00/0.00	0.00/0.00
14	CL13	Low complexity	62.59/0.14	0.00/0.00	0.00/0.00	0.00/0.00	0.00/0.00	0.00/0.00	0.05/0.00	19.01/0.04	0.00/0.00	0.00/0.00
15	CL15	TIR: CACTA	59.83/0.13	6.75/0.02	9.18/0.01	8.23/0.02	24.52/0.03	16.01/0.05	18.42/0.00	34.07/0.08	14.58/0.03	10.44/0.03
16	CL16	LTR: Copia	57.51/0.13	1.25/0.00	62.50/0.10	18.03/0.05	35.28/0.04	1.49/0.00	0.51/0.00	54.33/0.12	46.61/0.11	14.22/0.04
17	CL18	LTR: Gypsy	52.88/0.12	0.00/0.00	0.00/0.00	0.00/0.00	0.00/0.00	0.00/0.00	0.00/0.00	0.00/0.00	0.00/0.00	0.00/0.00
18	CL17	LTR: Copia	48.55/0.11	10.21/0.02	14.38/0.02	0.00/0.00	28.13/0.03	50.86/0.15	8.81/0.00	33.48/0.08	24.17/0.05	0.00/0.00
19	CL20	LTR: Gypsy	41.90/0.09	0.05/0.00	0.00/0.00	0.00/0.00	0.00/0.00	0.00/0.00	0.00/0.00	0.31/0.00	0.00/0.00	0.00/0.00
20	CL23	35S rDNA	40.26/0.09	40.66/0.09	103.17/0.16	46.89/0.14	95.21/0.11	25.03/0.07	54.34/0.01	62.42/0.14	67.77/0.15	29.40/0.08
21	CL21	LTR: Copia	38.70/0.08	8.11/0.02	26.53/0.04	8.26/0.02	21.23/0.02	10.66/0.03	14.82/0.00	55.94/0.13	31.81/0.07	0.00/0.00
22	CL19	LTR: Gypsy	38.49/0.08	0.02/0.00	0.00/0.00	0.00/0.00	0.00/0.00	0.00/0.00	0.00/0.00	5.10/0.01	0.00/0.00	0.00/0.00

23	CL22	LTR: Gypsy	35.95/0.08	0.03/0.00	0.00/0.00	0.00/0.00	0.00/0.00	2.79/0.01	0.35/0.00	30.76/0.07	0.91/0.00	0.00/0.00
24	CL24	LTR: Copia	35.59/0.08	0.01/0.00	17.09/0.03	0.00/0.00	53.48/0.06	41.90/0.12	0.00/0.00	30.55/0.07	20.45/0.05	0.00/0.00
25	CL25	LTR: Gypsy	31.25/0.07	4.49/0.01	5.83/0.01	19.16/0.06	0.15/0.00	9.22/0.03	5.85/0.00	8.18/0.02	17.46/0.04	0.77/0.00
26	CL27	Low complexity	30.82/0.07	0.00/0.00	0.00/0.00	0.00/0.00	0.00/0.00	0.00/0.00	0.00/0.00	1.94/0.00	0.00/0.00	0.00/0.00
27	CL28	Low complexity	30.35/0.07	0.00/0.00	0.00/0.00	0.00/0.00	0.00/0.00	0.00/0.00	0.00/0.00	0.00/0.00	0.00/0.00	0.00/0.00
28	CL26	Low complexity	26.57/0.06	0.00/0.00	0.00/0.00	0.00/0.00	0.00/0.00	0.00/0.00	0.00/0.00	0.00/0.00	0.00/0.00	0.00/0.00
29	CL29	LTR: Gypsy	24.88/0.05	0.07/0.00	0.00/0.00	0.00/0.00	0.00/0.00	0.07/0.00	0.05/0.00	5.23/0.01	0.07/0.00	0.00/0.00
30	CL30	LTR: Copia	24.36/0.05	0.00/0.00	48.33/0.08	0.00/0.00	32.26/0.04	4.35/0.01	0.00/0.00	23.22/0.05	28.81/0.07	0.77/0.00
31	CL34	LINE: L1	23.29/0.05	0.52/0.00	0.77/0.00	0.00/0.00	0.00/0.00	3.06/0.01	0.00/0.00	15.67/0.04	0.41/0.00	0.00/0.00
32	CL39	35S rDNA	22.90/0.05	32.36/0.07	92.01/0.14	56.05/0.17	76.98/0.09	35.69/0.11	56.95/0.01	44.68/0.10	56.34/0.13	36.55/0.10
33	CL31	LTR: Gypsy	21.65/0.05	0.00/0.00	0.00/0.00	0.00/0.00	0.00/0.00	0.00/0.00	0.00/0.00	0.00/0.00	0.00/0.00	0.00/0.00
34	CL32	LTR: Copia	21.59/0.05	0.75/0.00	0.00/0.00	0.00/0.00	0.00/0.00	0.00/0.00	0.00/0.00	0.22/0.00	0.00/0.00	0.94/0.00
35	CL37	Low complexity	21.20/0.05	0.26/0.00	0.00/0.00	0.05/0.00	0.70/0.00	0.96/0.00	0.00/0.00	0.00/0.00	0.78/0.00	0.00/0.00
36	CL33	LTR: Copia	21.13/0.05	1.09/0.00	26.64/0.04	0.00/0.00	4.38/0.01	4.90/0.01	0.67/0.00	32.74/0.08	17.40/0.04	7.50/0.02
37	CL35	Low complexity	21.07/0.05	0.00/0.00	0.00/0.00	0.00/0.00	0.00/0.00	0.00/0.00	0.00/0.00	8.13/0.02	0.00/0.00	0.00/0.00
38	CL36	LTR: Gypsy	19.86/0.04	0.00/0.00	0.00/0.00	0.00/0.00	0.00/0.00	0.00/0.00	0.00/0.00	15.16/0.03	0.00/0.00	0.06/0.00
39	CL38	Low complexity	19.81/0.04	0.00/0.00	5.94/0.01	0.00/0.00	0.00/0.00	0.00/0.00	0.00/0.00	1.17/0.00	0.00/0.00	0.00/0.00
40	CL40	Low complexity	19.49/0.04	0.00/0.00	0.00/0.00	0.00/0.00	0.00/0.00	0.00/0.00	0.00/0.00	9.18/0.02	0.00/0.00	0.08/0.00
41	CL41	LTR: Gypsy	19.47/0.04	0.01/0.00	0.00/0.00	0.00/0.00	0.00/0.00	0.00/0.00	0.00/0.00	0.00/0.00	0.00/0.00	0.00/0.00
**TOTAL**			3948.77/8.66	145.31/0.32	663.88/1.04	170.58/0.51	454.17/0.53	268.51/0.80	210.31/0.05	1398.10/3.22	523.74/1.19	101.33/0.27

*Clusters are ranked in order of their abundance in *F. imperialis*.

†Names from RepeatExplorer.

‡Given to 2 dp. Values for *F. imperialis* are shown first; other species are then listed alphabetically.

Table S7 Single/low-copy fraction size and genome size.

Species	Family	% single/low-copy DNA*	Ploidy†	1C-value (Mb)	Size of single/low-copy fraction per 1C genome‡	1Cx-value (Mb)	Size of single/low-copy fraction per 1Cx genome
Agoseris grandiflora	Asteraceae	57.01	2	1956	1115	1956	1115
Anacyclus depressus	Asteraceae	11.03	2	6064	669	6064	669
Anemone blanda	Ranunculaceae	42.99	2	14743	6338	14743	6338
Anemone coronaria	Ranunculaceae	47.01	n/a	10915	5131	n/a	n/a
Anemone cylindrica	Ranunculaceae	34.98	2	9095	3182	9095	3182
Anemone pavoniana	Ranunculaceae	38.00	2	12152	4618	12152	4618
Anemone riparia	Ranunculaceae	33.02	2	8753	2890	8753	2890
Anthemis altissima	Asteraceae	33.02	n/a	7726	2551	n/a	n/a
Anthemis montana	Asteraceae	36.99	n/a	8264	3057	n/a	n/a
Avena sativa§	Poaceae	21.00	6	12934	2716	4311	905
Beta vulgaris	Amaranthaceae	36.99	2	1223	452	1223	452
Brassica pekinensis (syn. *Brassica rapa* subsp. *pekinensis*)¶	Brassicaceae	47.01	2	782	368	782	368
Capsella bursa-pastoris	Brassicaceae	52.76	4	391	206	196	103
Cinnanomum camphora	Lauraceae	62.70	2	587	368	587	368
Crepis conyzifolia	Asteraceae	43.50	2	5389	2344	5389	2344
Crepis vesicaria	Asteraceae	25.98	2	4088	1062	4088	1062
Daucus carota	Apiaceae	38.00	2	978	372	978	372
Decaisnea fargesii	Loranthaceae	51.50	2	1980	1020	1980	1020
Glycine max§	Fabaceae	46.00	2	1100	506	1100	506
Gossypium hirsutum	Malvaceae	68.05	4	2347	1597	1174	799
Hordeum vulgare	Poaceae	30.00	2	5428	1628	5428	1628
Hyacinthus orientalis	Asparagaceae	24.98	2	20856	5210	20856	5210
Lactuca serriola	Asteraceae	44.99	2	1809	814	1809	814
Lamium purpureum	Lamiaceae	40.01	2	1076	430	1076	430
Lathyrus articulatus	Fabaceae	44.01	2	5941	2615	5941	2615

Lathyrus hirsutus	Fabaceae	30.02	2	9756	2929	9756	2929
Lathyrus nissolia	Fabaceae	41.00	2	6308	2587	6308	2587
Lathyrus ochrus	Fabaceae	40.01	2	6675	2671	6675	2671
Lathyrus sativus	Fabaceae	33.99	2	8215	2793	8215	2793
Linum usitatissimum	Linaceae	41.00	2	685	281	685	281
Liriodendron tulipifera	Magnoliaceae	52.49	2	782	411	782	411
Magnolia soulangiana	Magnoliaceae	60.51	4	5844	3536	2922	1768
Matthiola incana	Brassicaceae	30.99	2	2064	639	2064	639
Microseris bigelovii	Asteraceae	48.51	2	1467	712	1467	712
Microseris douglasii	Asteraceae	54.00	2	1174	634	1174	634
Microseris laciniata	Asteraceae	46.00	2	3276	1507	3276	1507
Microseris lindleyi	Asteraceae	44.99	2	1956	880	1956	880
Nicotiana tabacum§	Solanaceae	45.00	4	5061	2277	2531	1139
Petroselinum sativum§ (syn. *Petroselinum crispum*)	Apiaceae	30.01	n/a	2201	660	n/a	n/a
Pinus strobus	Pinaceae	14.00	2	25086	3512	25086	3512
Pisum sativum§	Fabaceae	27.49	2	4768	1311	4768	1311
Poa trivialis	Poaceae	18.00	2	2763	497	2763	497
Pyrrhopappus carolianus	Asteraceae	38.00	2	6137	2332	6137	2332
Pyrrhopappus multicaulis	Asteraceae	32.02	2	6504	2082	6504	2082
Raphanus sativus	Brassicaceae	82.01	2	538	441	538	441
Secale cereale§	Poaceae	27.40	2	8093	2218	8093	2218
Senecio vulgaris	Asteraceae	25.98	4	1540	400	770	200
Spinacia oleracea	Amaranthaceae	44.99	2	1002	451	1002	451
Stellaria media	Caryophyllaceae	30.99	7	1027	318	293	91
Triticum aestivum§	Poaceae	21.00	6	16944	3558	5648	1186
Tropaeolum majus	Tropaeolaceae	18.03	4	1296	234	648	117
Tulipa kaufmanniana	Liliaceae	27.00	2	22078	5962	22078	5962
Veronica persica	Plantaginaceae	36.99	4	758	280	379	140
Vicia faba	Fabaceae	20.00	2	13032	2606	13032	2606

Vicia sativa	Fabaceae	20.00	2	2201	440	2201	440
Vigna radiata[§]	Fabaceae	50.01	n/a	513	257	n/a	n/a
Zea mays	Poaceae	21.94	2	2665	585	2665	585

[*]Data on the percentage of single/low-copy DNA were taken from Elsik & Williams (2000), Thompson (1978) and Wenzel & Hemleben (1982).

[†]1C-values and ploidy information were taken from release 6.0 of the Plant DNA C-values Database; genome sizes are given to the nearest Mb. For species where the Plant DNA C-values Database contains entries for individuals of different ploidy the diploid values were used. n/a denotes species where there is no ploidy information associated with the genome size estimate in the Plant DNA C-values Database.

[‡]The size of the S/L fraction per 1C and 1Cx genome size is given to the nearest Mb.

[§]Multiple independent estimates of the % of S/L DNA were available (three estimates for *Glycine max*, two estimates for all other species indicated), therefore, an average of all values was used.

[¶]Species are listed under the names given in the original papers, but where a different synonym is used in the Plant DNA C-values Database this is noted in parentheses.

Notes S1 Potential impact of differing sequence similarity thresholds on patterns of repeat diversity

A potential cause of contrasting patterns of repeat diversity between species is the application of different levels of stringency when delimiting families of repetitive elements and assessing their abundance in the genome. Nevertheless, there is no universal consensus on the threshold of sequence similarity that should be used when defining repeat families. A unified classification system for transposable elements was proposed by Wicker et al. (2007), who stipulated that in order to be classified within the same family, sequences must match within the coding region, internal domain or terminal repeat region for at least 80bp with a minimum of 80% similarity along 80% of the matching region (the “80-80-80 rule”). This system has been criticised recently by Elbaidouri and Panaud (2013) who suggest that it may lead to an over-estimation of the number of repeat families and an under-estimation of the abundance of individual families. Elbaidouri and Panaud (2013) propose an alternative approach for classification, albeit one that pertains only to long terminal repeat (LTR) retrotransposons, whereby two LTR retrotransposons belong to the same family if they have a minimum of 60% similarity over 70% of their LTR length.

Studies in species whose genomes are reported to be dominated by a small number of high abundance repeats have also used widely varying levels of stringency when delimiting repeat families and estimating their abundance. In their study of genome size evolution in *Oryza australiensis*, Piegu et al. (2006) assembled reference sequences for three LTR repeat families (which together were estimated to comprise 60% of the *O. australiensis* genome) by creating seed contigs from sets of ≥ 200 BAC-end sequences (BES) which had at least 95% similarity across the entire length of their alignment; further BES were then assembled with these seed contigs, using a cut off of at least 90% similarity across their overlapping regions. The copy number of each family (as well as the number of Mb they contributed to the genome) was then estimated using dot-blot hybridisation, at a stringency that is equivalent to *c.* 88% similarity (assuming a 45% GC content for the *O. australiensis* genome) between the probe and the target sequence across the full length of the probe (various probes were used, the sequences of which were not specified, but at least one probe of > 1000bp was used; Piegu et al. 2006). In contrast with the relatively high level of similarity across

comparatively long stretches of sequence required by Piegu et al. (2006), Hawkins et al. (2006) in their study of genome size evolution in cotton species, considered sequences to belong to the same repeat family if they had > 80% similarity over a region of at least 100bp. The whole genome shotgun sequences they used were on average > 700bp (Hawkins et al. 2006) meaning that sequences matching by > 80% over < 15% of the length would be assigned to the same family. Finally, in a study of the genome composition of barley (*Hordeum vulgare*) using 454 sequences with an average length of 103 bp, the abundance of known repeat families was estimated by using hit numbers from a BLAST search of the 454 reads against a database of reference sequences performed with an E-value cut off of 1×10^{-6}; any read matching one of the repeat family reference sequences with an E-value of $\leq 1 \times 10^{-6}$ was assigned to that family (Wicker et al. 2009). However, query sequences with different lengths can have the same percentage similarity and overlap with a subject sequence but different E-values, making it difficult to relate the level of stringency imposed by the use of a particular E-value to that applied in studies that have used a given level of sequence similarity as their cut off.

In our analysis of *Fritillaria*, read pairs were required to have ≥ 90% similarity across ≥ 55% of their length (equivalent to a minimum matching length of 220bp for the 400bp reads used during clustering) in order to belong to the same repeat family. When estimating the abundance of individual repeat families, reads had to match one of the reference contigs with ≥ 90% similarity across ≥ 55% of the read length in order to be assigned to a particular family. Consequently, the level of stringency applied during our analysis was higher than used in some previous studies and could result in more, lower abundance, repeat families being inferred than has been the case in other species. To test whether our approach to *de novo* identification and quantification of repeat families may create a false impression of higher diversity in *Fritillaria* than in other species, we used the same methods to analyse data from barley (*Hordeum vulgare*). We selected barley for this analysis because: 1 – previous data indicate its genomic composition contrasts starkly with that inferred for *Fritillaria*, as a large portion of the barley genome is made of a small number of high abundance repeat families (Wicker et al. 2009); 2 – data that are equivalent to those used in *Fritillaria* are available (i.e. 454 reads from the GS FLX Titanium platform), therefore removing the possibility that any

difference in the pattern of repeats between barley and *Fritillaria* is due to the use of different types of data. We downloaded a set of barley 454 reads from the Sequence Read Archive (SRA accession number ERR127132) and processed the reads to remove exact duplicates and organellar reads in the same way as described for *Fritillaria* (see Materials and Methods in main text) to create a set of unique nuclear reads for barley. The unique nuclear barley reads were then trimmed and filtered by length (see Materials and Methods) to create a set of 400bp reads. From this dataset, 100,332 reads were randomly sampled using the sequence sampling tool (v. 1.0.0) in RepeatExplorer to create a dataset providing the same level of genome coverage as used for *Fritillaria* (i.e. 0.74%; we used a genome size of 1C = 5.428 Gb for barley, which is the prime value for this species in the Plant DNA C-values Database release 6.0, http://data.kew.org/cvalues/). We then used RepeatExplorer to cluster the random sample of barley reads, with the same parameter settings as used for *Fritillaria*; cluster merger and the estimation of GP for each cluster were also carried out in the same way as described for *Fritillaria*. Repeat families were annotated with the results of a BLASTN search to the total TREP database (http://wheat.pw.usda.gov/ITMI/Repeats/) to allow direct comparison of our results with those of Wicker et al. (2009); the search was performed using an E-value cut off of 1×10^{-6} and clusters were annotated as the repeat type hit by the majority of contigs.

Comparison of the clustering results from barley and *Fritillaria* demonstrate that, at the same level of genome coverage (0.74%), a much higher percentage of reads can be clustered for barley than is the case for either of the *Fritillaria* species; 67219/100332 input reads (67.00%) were clustered for barley, compared with only 326887/830674 reads (39.35%) for *F. affinis* and 279426/842670 reads (33.16%) for *F. imperialis*. The total number of clusters identified in barley (following cluster merger) was only 4483, with an order of magnitude more clusters found in *F. affinis* (49989 clusters in total) and *F. imperialis* (71218 clusters in total). Moreover, whilst the top ten most abundant clusters account for only 17.63% and 6.08% of the *F. affinis* and *F. imperialis* genomes respectively, the top ten clusters from barley account for 38.17% of its genome. These results confirm that barley and *Fritillaria* have contrasting patterns of repeat diversity. We also compared our *de novo* estimates of repeat abundance in barley with those previously reported by Wicker et al. (2009). The most abundant clusters

identified via our approach match the most abundant repeats detected previously in the barley genome. The top five families (all LTR retrotransposons belonging to either the Copia or the Gypsy superfamily; Wicker et al. 2007) in both analyses are: Bare1 (Copia), Sabrina (Gypsy), Wham (Gypsy), BAGY2 (Gypsy) and Surya/Sukkula (Gypsy – the fifth ranked cluster from our analysis had a significant number of hits to both families; 64% of contigs had a top hit to Surya and 36% of contigs had a top hit to Sukkula). Whilst Wicker et al. (2009) estimated that the top five repeat families in barley accounted for 35.38% of the genome, the abundance calculated via our approach is slightly lower at 30.33%. Also, our abundance estimates for four out of five of the top repeat families are slightly lower than those calculated by Wicker et al. (2009; Bare1 - 9.97% vs. 12.69%; Sabrina - 7.40% vs. 8.45%; Wham - 5.34% vs. 5.50%; Surya/Sukkula - 2.37% vs. 3.59%), although we estimate a higher abundance for the BAGY2 family (5.25% in our analysis versus 5.15% in Wicker *et al.* 2009). Although Bare1 was identified as the most abundant repeat family in both our analysis and that of Wicker *et al.* (2009), we also identified another repeat family with similarity to Bare1 (ranked eighth most abundant in the barley genome, with a genome proportion of 2.08%). We did not to merge the two Bare1-type clusters, as both already contained a complete set of conserved domains and although they formed connected components the proportion of similarity hits shared between the two clusters was relatively low (data not shown). However, the combined abundance of these two clusters approaches that estimated previously for Bare1 (*c.* 12% vs. 12.69% estimated by Wicker et al. 2009).

The comparison between barley and *Fritillaria* illustrates that our approach to *de novo* repeat family identification and quantification might result in some additional families being recognised, with consequently lower abundance for individual families, compared with the results of previous studies. Nevertheless, it is also clear that any difference in stringency between the methods we have used and those that have been applied elsewhere does not change the overall picture of repeat diversity in the species analysed. Applying our approach to the analysis of data from barley still reveals a large fraction of the genome to be comprised of a small number of high abundance repeats. The result from this test shows that differences in the specific methods for characterizing repeats are not responsible for creating the broad-scale differences in the patterns of repeat diversity detected, and instead the contrasting genomic composition of *F. affinis* and *F.*

imperialis versus plants with smaller genomes reflect real difference in the biology of these species.

Notes S2 Analysis of intra-family heterogeneity of repeats in *Fritillaria*

Analysis of the repeat content of *Fritillaria* demonstrates that lineage specific genome size increases cannot be accounted for by the amplification of just a few repetitive element families, as shown in some other plant groups (Hawkins et al. 2006; Piegu et al. 2006). Moreover, the bulk of the genomes of *F. affinis* and *F. imperialis* are apparently composed of a diverse set of relatively low abundance repetitive/repeat-derived DNA. This high level of heterogeneity within the repetitive fraction of the genome could have arisen via distinct pathways: 1 – global amplification of repetitive DNA and high genome turnover, so that many repeat families amplify simultaneously but remain relatively small in size due to rapid deletion of amplified copies; 2 – simultaneous amplification of a number of different repeat families accompanied by low rates of deletion, so that amplified copies accumulate in the genome creating an increasing fraction of repeat-derived DNA that degenerates and diverges over time. If the first of these scenarios is responsible for the pattern of repeat diversity seen in *Fritillaria* then we would expect individual repeat families to be dominated by recently amplified copies that have a high level of sequence similarity to each other. By contrast, if the second of these scenarios were true then repeat families would be predicted to contain copies that had amplified at different times and therefore show greater levels of divergence from one another.

To analyse the level of heterogeneity within individual *F. affinis* and *F. imperialis* repeat families identified from the RepeatExplorer analysis, we examined the average edge weights for graphs from all clusters that include ≥ 0.05% of the input reads (i.e. the top repeat families; see Table S5, S6). Edge weights are determined using similarity scores from the megablast step of the RepeatExplorer analysis (Novák *et al.,* 2013); higher levels of overlap and sequence similarity between read pairs result in higher edge weights. Therefore, clusters with a higher average edge weight contain a larger number of high similarity pairs than clusters with lower average edge weights. The majority of the top repeat families from *F. affinis* and *F. imperialis* have graphs with average edge weights of < 450, with a small number having values of ≥ 500 (Fig. 3

in main manuscript). For individual repeat families with a range of different average edge weights, we performed all versus all BLAST searches of their constituent reads and recorded pair-wise similarities for hits passing a threshold of ≥ 55% overlap between the query and subject sequence with ≥ 90% similarity in the overlapping region. BLAST searches were performed with the same parameter settings as for the *de novo* identification of repeat families (see Materials and Methods in main text). A custom Perl script was then used to filter out hits that did not pass the similarity threshold; self hits and reciprocal hits were also removed. For the filtered set of BLAST hits, histograms of the percentage similarities between read pairs from individual clusters were generated in R (Fig. 3). Plots of sequence similarity for repeat families with average edge weights of < 450 show an absence of peaks of very high similarity read pairs (i.e. ≥ 98% sequence similarity; see plots for representative families in Fig. 3), with the majority of read pairs having < 95% sequence similarity. Although there are a small number of highly similar read pairs, suggesting recent amplification, the pattern of sequence similarity in these repeat families is indicative of the accumulation of copies over time, resulting in read pairs with differing levels of divergence (e.g. Fig. 3c,g). By contrast, plotting the pair-wise sequence similarities for representatives of those few repeat families whose graphs have average edge weights > 500 reveals that they are predominantly composed of reads with high (≥ 98%) similarity to each other, indicative of recent amplification and/or homogenization (Fig. 3e,j).

Supplementary References

Ambrožová K, Mandáková T, Bureš P, Neumann P, Leitch IJ, Koblížková A, Macas J, Lysák MA. 2011. Diverse retrotransposon families and an AT-rich satellite DNA revealed in giant genomes of *Fritillaria* lilies. *Annals of Botany* **107:** 255–268.

Day PD, Berger M, Hill L, Fay MF, Leitch AR, Leitch IJ, Kelly LJ. 2014. Evolutionary relationships in the medicinally important genus *Fritillaria* L. (Liliaceae). *Molecular Phylogenetics and Evolution* **80:** 11–19.

Doležel J, Bartoš J, Voglmayr H, Greilhuber J. 2003. Nuclear DNA content and genome size of trout and human. *Cytometry A* **51:** 127–128.

Elbaidouri M, Panaud O. 2013. Comparative genomic paleontology across plant kingdom reveals the dynamics of TE-driven genome evolution. *Genome Biology and Evolution* **5:** 954–965.

Elsik CG, Williams CG. 2000. Retroelements contribute to the excess low-copy number DNA in pine. *Molecular and General Genetics* **264:** 47–55.

Fujimoto S, Ito M, Matsunaga S, Fukui K. 2005. An upper limit of the ratio of DNA volume to nuclear volume exists in plants. *Genes Genet Syst*, **80:** 345–350.

Greilhuber J, Doležel J, Lysak MA, Bennett MD. 2005. The origin, evolution and proposed stabilization of the terms 'Genome Size' and 'C-Value' to describe nuclear DNA contents. *Annals of Botany* **95:** 91–98.

Hanson L, McMahon KA, Johnson MAT, Bennett MD. 2001. First nuclear DNA C-values for 25 angiosperm families. *Annals of Botany* **87:** 251–258.

Hawkins JS, Kim HR, Nason JD, Wing RA, Wendel JF. 2006. Differential lineage-specific amplification of transposable elements is responsible for genome size variation in *Gossypium*. *Genome Research* **16:** 1252–1261.

Leitch IJ, Beaulieu JM, Cheung K, Hanson L, Lysak MA, Fay MF. 2007. Punctuated genome size evolution in Liliaceae. *Journal of Evolutionary Biology* **20:** 2296–2308.

Novák P, Neumann P, Pech J, Steinhaisl J, Macas J. 2013. RepeatExplorer: a Galaxy-based web server for genome-wide characterization of eukaryotic repetitive elements from next-generation sequence reads. *Bioinformatics* **29:** 792–793.

Piegu B, Guyot R, Picault N, Roulin A, Saniyal A, Kim H, Collura K, Brar DS, Jackson S, Wing RA, *et al.* 2006. Doubling genome size without polyploidization: dynamics of retrotransposition-driven genomic expansions in *Oryza australiensis*, a wild relative of rice. *Genome Research* **16:** 1262–1269.

Thompson W. 1978. Perspectives on the evolution of plant DNA. *Carnegie Institute of Washington Year Book* **77:** 310–316.

Wenzel W, Hemleben V. 1982. A comparative study of genomes in angiosperms. *Plant Systematics and Evolution* **139:** 209–227.

Wicker T, Sabot F, Hua-Van A, Bennetzen JL, Capy P, Chalhoub B, Flavell A, Leroy P, Morgante M, Panaud O, Paux E, SanMiguel P, Schulman AH. 2007. A unified classification system for eukaryotic transposable elements. *Nature Reviews Genetics* **8:** 973–982.

Wicker T, Taudien S, Houben A, Keller B, Graner A, Platzer M, Stein N. 2009. A whole-genome snapshot of 454 sequences exposes the composition of the barley genome and provides evidence for parallel evolution of genome size in wheat and barley. *Plant Journal* **59:** 712–722.
